# Renewable solar and wind energies on buildings for green ports in Egypt

**DOI:** 10.1007/s11356-023-25403-z

**Published:** 2023-02-06

**Authors:** Magdy Tawfik, Ahmed S. Shehata, Amr Ali Hassan, Mohamed A. Kotb

**Affiliations:** 1 Marine and Offshore Engineering Department, College of Engineering and Technology, Arab Academy for Science Technology and Maritime Transport, B.O. Box 1029 Alexandria, Egypt; 2 Marine Engineering and Naval Architecture Department, Alexandria University, Alexandria, P.O. 21544 Egypt

**Keywords:** Solar energy, Wind energy, Numerical approaches, Energy management, Egyptian green ports, Environmental pollutions

## Abstract

Energy management plan is utilized as an optimum strategy by using solar and wind energies, as a new preliminary implementation. The aim of the study is to create an optimum strategy through an optimization of an energy management system. The study implemented an onsite model, two numerical approaches, and an optimization analysis on a Mediterranean port. Two approaches have been used: solar energy is applied experimentally and numerically, and then wind energy is simulated. An optimization analysis integrated the two approaches together to control their operation. The results showed the installed solar panels provided sufficient generated power for the buildings. Also, the simulated wind arrays showed good behavior with increased power coefficient for the wind turbines, for future implementation. These results were validated using the DesignBuilder software and showed accurate values regarding the experiment for solar panels and CFD simulation. Eventually, a Pareto optimality analysis is applied between the solar and wind energies to reveal an energy management plan. Renewable energy offered energy to support the consumption of the port’s buildings.

## Introduction

Economic globalization is arising as the crucial role of maritime transportation, throughout port industries. Ports are accessible gates for the global networks that could be an indicator of economic performance. Those logistics platforms have caused a wide urban expansion, which integrates the high-density harbor front areas. The concept of port developments has become one of the most competitive among worldwide ports. Integrations of the port’s systems improve this competitiveness in the global maritime supply chain. Despite the growing ports, strategies are set to reduce emissions in shipping and ports.

Ports are a major emitter of environmental pollutants and noises, to both marine and ecosystems. If the port is located within the city limits, environmental factors should be considered when evaluating port efficiency. Shipping accounts for environmental impacts such as GHG emissions (Tovar and Wall 2019). Greenhouse gas (GHG) is one of the root causes of climate change and global warming (Tovar and Wall 2019). The main sources also inside the port are cargo loaders, such as trucks, trains, ships, and pipelines. The port industry has a great desire toward mitigating climate change as a target of environmental protection (Iris and Lam 2019).

Few port authorities have actively pursued energy management strategies (Acciaro et al. 2014). Comparing the competitive developments among ports, independent power producers at the port have their own benefits over the dependent ports. Several innovative technologies of operational strategies and energy usage patterns are obtained to enhance zero-energy ports. In the order of investigation of the green port’s conversion, several perspectives are undertaken into considerations, including the port’s characteristics, best practices, limitations, opportunities, and key issues (Alamoush et al. 2022).

## Background

Several studies have tackled the emissions of ports and the conversion into green seaports. Some of these studies were numerically, as follows.

The researchers in Chang and Wang (2012) numerically studied an energy management model to reduce port emissions for green port conversion. The results showed that reducing the ship’s speed approaching the berth to 12 knots could reduce the emissions. However, using the onshore power supply could reduce the CO_2_ emission by 57.16% and PM by 39.4%. Decreasing the ship’s speed by policies at a range of 20 nautical miles, as well as using the cold ironing, would cut down the emissions by 71 to 91%. Hou and Geerlings (2016) numerically studied the sustainability measures and their effectiveness through a conceptual framework in the port of Shanghai. The study focused on both the qualitative and the quantitative outputs. The results concluded that the process has to be organized as well; new policies should be produced to control the transition.

Then, Chang and Jhang (2016) numerically conducted a green flag program to specify the emission reductions caused by the bulk and container ships entering the port of Kaohsiung. That model is used to account for fuel consumption as well the emissions when using a lowered speed of 12 knots at 20 nautical miles. Also, that model can be used when the speed of ships are lowered to 12 knots and 20 nautical miles far from the port. The results showed a CO_2_ emission reduction of 41% and 14% for the container and bulk vessels, respectively. Meanwhile, the SO_2_ emission reduction rates are 48% and 43% for the container and bulk vessels, respectively. The results showed that large-sized ships produce less emission, while container ships adopted low speed and fuel transportation, unlike bulk carriers. Kotrikla et al. (2017) numerically estimated the particle matter and the CO_2_ emission values emitted by ships at the port using the bottom-up methodology. Also, they investigated the shore connection possibilities to reduce those emissions. The study investigated renewable energy applications by using the HOMER Energy microgrid simulation software. The study showed that 40 ships in the port of Mytilene during 10 days produced 441 kg of PM and 282 metric tons of CO_2_. Around 63% and 77% of PM and CO_2_ are emitted by the maneuverability at the berthing phase only. The study concluded that emissions could be avoided by using solar wind renewable system.

Tovar and Wall (2019) numerically accounted for the environmental efficiency of 28 Spanish port authorities in 2016. The nonparametric program of data envelopment analysis (DEA) is used to measure the CO_2_ emissions by an output-oriented directional distance frontier. The novel dataset showed that environmental efficiency could improve the CO_2_ emission by 63%. Controlling the cargo and passenger traffic achieves an emission reduction of up to 82%. Wang et al. (2020) investigated the maritime port developments using a numerical regression model. The study has provided the intragroup cooperation and out-group competition development model. The results showed that green efficiency model evaluated through 18 ports in China’s five-port groups is compared from 2012 to 2016 using the data envelopment analysis. The study found low efficiency of Chinese ports, due to the numerous problems existing in developing the ports, while the proposed model would improve the ports and reduce the emissions.

Song et al. (2020) numerically implemented a comparative study of 5 scenarios to find the best minimum planning cost while investigating the daily operating conditions. The study showed a framework for modeling an integrated port energy system. Also, the study conducted an analysis on the ships using shoreside power on planning costs. The study showed the effectiveness of the port with different energy infrastructures, as well as the planning cost decreasing significantly. Hua et al. (2020) numerically suggested and studied the green port indicator system tailored-made at the Zhuhai Port. The study used a fuzzy importance-performance analysis (FIPA) method to find out the port’s performance. The results indicated that the port has to overview and monitor its energy consumption, pollutant emissions, applied technologies, and development. Those observations could be the concept of green development at the port’s facilities.

Dan et al. (2013) statistically simulated the efficiency of coastal container terminals in China. A three-stage data envelopment analysis model is used for 42 coastal container terminals, which aimed to find higher port efficiency and an improved development mode. The results revealed that efficiencies of coastal container terminals are lower than those of the other ports; however, the scale inefficiency is the primary reason, which is causing a big difference of different port groups. The results showed excessive waste in the input phases. Hanssen et al. (2014) investigated the possibilities of applying bio-solar energy systems in the Netherlands. The study aims to use the renewable method to produce clean fuel and reduce CO_2_ emissions. The conducted study evaluated two technological features concerning the energy port and energy farm, which are introduced at workshops of researchers and policymakers. Gibbs et al. (2014) analyzed the port’s data to reduce the greenhouse gases, especially in the UK. The provided data are collected from reports and interviews. This analysis showed that the amount of emission produced by the ship at sea is greater than that of their counterpart at ports. However, the ship emission would be reduced; the port’s emission is still high and could be reduced through different plans. Acciaro et al. (2014) analyzed the energy flows of two ports (Hamburg and Genoa) to investigate their energy flow.

The results showed that the proposed energy management plans could provide substantial efficiency gains. This in turn contributes to the development of new alternative revenue sources, which enhance the competitive position of ports.

Huertas-Fernández et al. (2021) theoretically and numerically introduced and analyzed a novel method to use the wave energy converters as the oscillating water column to produce green hydrogen gas. Hydrogen gas could be used as a clean source of fuel for the energy management strategy. Simulation was implemented using a dynamic financial index and Monte Carlo techniques to determine the repayment period, as well the performance of the power plant for the port of Motril, Southern Spain. The results indicated that it can provide sufficient power for the repayment period. Damman and Steen (2021) implemented a multi-level perspective (MLP) to investigate three Norwegian ports to figure out zero-emission energy hubs. The study also showed the importance to be a green port by using different disciplines such as hydrogen and LNG. That study revealed that there is an interaction between geographical factors and institutional influences, which could lead to new solutions, feedback, and contributions toward green ports.

Abdeen et al. (2019) simulated several geometric parameters to investigate heat transfer processes and airflow in a Trombe wall during winter in Alexandria, Egypt, using the DesignBuilder software. While the Trombe walls have massive natural convection and energy storage capability, solar energy is used for decreasing the heating load. The results showed the optimal design of the Trombe wall has a height of 1.7 m by a thickness of 0.3 m and a channel depth of 0.22 m, which enhances the thermal comfort by 38.19% during winter. Fouad et al. (2020) modeled 8 building designs with 52 buildings using the DesignBuilder and EnergyPlus software to investigate a zero-energy city, in Egypt. The study results showed that renewable energy achieved reduction by 57.6% of the energy consumption, with CO_2_ reduction by 390 tons. The study also may help policymakers and governments to achieve zero-energy cities.

Roos and Neto (2017) exploratory investigated the environmental performance of Brazilian ports by two state agencies and data of two port authorities. The study found the environmental management should be consolidated before any advanced environmental discussions. Laxe et al. (2017) investigated the sustainability synthetic indexes on a specific port in Spain; however, one simple consideration is used such as the ecological footprint. The study showed that, unlike the local synthetics, the global synthetics are a multidimensional nature that uses economic, institutional, environmental, and social dimensions to investigate the port area. Arena et al. (2018) utilized a pilot feasibility analysis of integrating green energy, which was applied to passengers and freight mobility near the Italian port area. The utilized sea-to-grid system that provides electricity by harvesting the sea wave energy, as well the green system, would use fully electric vehicles (FEVs) in and close to the port area. The results showed that the energy-producing technology cut down the emissions. The sea-to-grid system provides sufficient electrical power to the green system that leads to experimentation of medium size urbanized areas to small size ports.

Aregall et al. (2018) reviewed any possibilities to improve the environmental performance of ports across the globe. The results indicated that 76 out of 365 ports were examined. Several major measures are applied, and three are found as the most common, such as technology improvements, infrastructure development, and monitoring programs. Hentschel et al. (2018) investigated the design and application of renewable energy cooperatives at the Port of Rotterdam. The study revealed an action plan to precede a successful cooperative at the port, which could be a guide for stakeholders. Di Vaio and Varriale (2018) implemented a numerical qualitative method for the Italian seaports to assess the managerial key performance indicators. The study’s aim is to reduce the environmental impact and energy effects from ports. The results revealed a decision-making tool to achieve high competitiveness of the port that supports interorganizational relationships with shipping lines.

Sun et al. (2017) evaluated and analyzed the efficiency of Chinese ports. The study used a proposed model that implemented a nonradial (data envelopment analysis) preference model and used some assumptions to carry out the analysis. The study output showed that the efficiency was low for all ports when environmental factors were considered. It also showed that the port assets, berth quantity, and geographical location can significantly impact environmental performance. Nebot et al. (2017) assessed 6 strategies for improving the ports’ energy management. The strategies are used before in different discipline works, such as the marine strategy and integrated coastal management, sustainable port infrastructures, port networking initiatives, regionalization of ports, urban and landscape connectivity, and social integration of ports.

Castellano et al. (2020) used a multistep strategy to evaluate an environmental management system. Two indicators are introduced, i.e., environmental quality and port authorities’ efforts, based on 24 Italian ports in 2016. Then, the port efficiency-related economic efficiency is used to enhance the environmental management system. The study found that a used framework is crucial for ports’ environmental and economic performance and to avoid any partial judgments. Also, the system has been optimized by the adoption of green port policies, which use economic performance and minimize undesirable output. The implemented proactive green policies achieved the efficiency converges.

Lee and Nam (2017) analyzed the green shipping market to assess the benefits of each stakeholder in major countries. The system also aims to study the eco-friendly vessels and the involved regulations, affected by the shipping companies, shipyards, ports, and policies. The results introduced strategic plans towards green shipping in South Korea, such as initiating a shipping-shipbuilding collaborative network, sharing information, increasing the investment in R&D, and using an advanced fueled ship.

Poulsen et al. (2018) studied the limitations and any possible future potential of initiatives to enhance environmental management. That analysis found from the global chains that low tool implementation complexity and high emission visibility are crucial for recovering the environment. The results showed two major ways to improve the environmental performance of maritime ports. Firstly, it could be achieved by increasing the collaboration between global chains to lower the implemented tool complexity. Secondly, it should enhance emission visibility by cargo owners and regulators. Oh et al. (2018) investigated the port’s sustainability to specify the crucial criteria in South Korea. The study used the importance-performance analysis technique to assess the sustainability-environmental, economic, and social aspects by 27 sustainability items. The study findings showed that the most important factor is economic issues followed by environmental concerns and then social factors. Through interviews, port managerial insights are explained to provide more improvement areas to enhance port competitiveness.

Hossain et al. (2019) evaluated the sustainability and environmental performance of 18 Canadian major ports. The study has undertaken twenty-five pre-defined indicators of the operational trends to investigate its sustainability. Also, the study analyzed annual performance reports of all Canadian major ports to assess several pollutant performances. The study showed that all Canadian ports have to participate in the annual performance report and use the proposed initiatives to enhance their overall environmental performance. Li et al. (2019) optimized numerically the installation and operation strategy of a hybrid renewable energy system especially offshore wind energy for container terminals in Northeast China. The mixed-integer optimization model is used and has concerned with maximizing environmental and economic benefits. That optimization model is considered with two objectives, with several constraints, such as energy supply, demand balances, energy storage balances, technical constraints, and quantity limitations. The model was obtained to specify the hourly energy demands in the terminals, under three evaluation strategies, such as cost-saving, low-carbon, and trade-off strategy. The results revealed that the optimization algorithm would provide references for designing and construction policy of green container terminals.

Martínez-Moya et al. (2019) investigated the energy consumption and CO_2_ emissions of the container terminals of Valencia, Spain. The study showed that the yard had a large value of CO_2_ emissions at 68.1%, as the tractors and rubber-tired gantry cranes (RTGs) ran. The energy efficiency would be improved by using a new liquefied natural gas tractor to cut down the emissions. Policy implementation by the port authorities may mitigate the terminal emissions too. Gutierrez-Romero et al. (2019) implemented a numerical study on the data port traffic in the period 2010–2016 at Cartagena port, Spain. This study predicted the total energy consumption data used at the port, which is provided by the port authority. The Monte Carlo technique is used to obtain the hourly energy consumption, to specify renewable energy resources to be used. The energy demand relies on the solar, onshore, and offshore wind resources available in the Cartagena area. Also, the study found that solar resource is not sufficient, unlike wind resource. The results highlight that the use of renewable energy resources can reduce more than 10,000 tons of CO2 annually in addition to the greenhouse gases and other pollutant gases as well. Tsai et al. (2018) controlled the greenhouse gas (GHG) and emissions using a self-management approach, in port Taichung. The year 2014 is considered the basis year to be a reference of emissions. The results showed that the development of a green port is applicable to protect the environment, as it is used in other industries and areas.

Peng et al. (2019) studied and determined a strategy of onshore power supply to power up the ships at berth. The methodology aims to reduce the carbon emissions of ships as well decreasing the trade-off cost. Also, the study numerically optimized the operating system in a container terminal at a port in China. Changing the electricity prices affects the sensitivity of electricity prices. Iris and Lam (2019) investigated different innovative technology that could be applied to achieve a low-carbon port model. The proposed technologies include operational strategies, technology usage, renewable energy, alternative fuels, and energy management systems. The results indicated that many worldwide ports seek to increase their ability to be converted to green ports, while there is few ports application available. Teerawattana and Yang (2019) applied an entropy analysis on the environmental performance indicators (EPIs) of the Laem Chabang port, in Thailand, using data from 2011 to 2014. That analysis aimed to figure out the ports assessment criteria to be converted into green maritime ports. The results revealed that the most crucial aspects to be underlined are the wastewater, chromium in soil and sediment, pollutants in the air, phytoplankton biodiversity, and zooplankton biodiversity.

Alamoush et al. (2022) differentiated between technical and operational measures, while implementation a conceptual framework of policymakers, polluters, GHG emission, and operational measures using schemes. The study applied to identify policy instruments and to reduce GHG emissions, by the maritime sector. The results showed that different implementations should be taken into considerations to reduce polluters. Those measures are introduced in monitoring of emissions as the best implementation schemes by port policymakers and public or port authorities that suggested measures to cut down the (GHG) emissions. Also, Bergqvist and Monios (2019), Du et al. (2019), He (2018), and Pallis and Vaggelas (2019) have explained more in their book chapters. On the other side, buildings are substantial energy consumers worldwide. In this context, energy audit optimizes the complexity of building operation thermal, energy, and economic performance. Zero-energy building is spreading rapidly reaching to the reduction of energy consumption and carbon emissions, while achieving the associated comfort. More specifically, one of the most powerful simulation tools is the DesignBuilder software. Few studies pay close attention to the operational performance of buildings.

Alamoush et al. (2020) carried out a review of the port’s technical and operational measures and their greenhouse gas emission. The study categorized the ports’ measures in order to reduce and improve energy efficiency. The results showed that any single measure could lead to port decarbonization to varying abatement potential, complexity, and cost. Sifakis and Tsoutsos (2021) reviewed scientific literature and any viable solutions to find zero-energy ports worldwide. The reviewed measures are analyzed to specify the pros and cons for any future implementation to improve the port’s economic effectiveness. The study investigated the gaps between ports according to their strengths, weaknesses, opportunities, and threats to categorize ports to their specific characteristics. Also, it could be used as a port decision-maker and researcher tool to be a viable solution. The results showed that the majority of green energy generation technologies’ energy management strategies are still under investigation. Molavi et al. (2020) proposed a systematic framework to investigate microgrid integration to create a sustainable application. The two-stage mixed-integer optimization model is used to enhance the port performance. The results showed that the framework could guarantee the port operations to be improved in productivity, sustainability, and reliability.

Mokhtara et al. (2021) conducted simulations to find out the optimal design of a hybrid renewable energy system. That system consists of diesel, PV, wind, and battery packs to support residential buildings in rural areas, in Algeria. Multicriteria spatial analysis is used to describe the renewable energy potential map. After that, the particle swarm optimization algorithm optimized the multi-objective problem, whereas the main objective is minimizing the cost of energy (COE) and maximizing system reliability with the renewable fraction (RF). The results showed that the multi-criteria spatial analysis identified seven locations at seven zones. As well, the particle swarm algorithm revealed two locations to be utilized/powered by the photovoltaic, wind, diesel, and battery, when 5 locations by the photovoltaic, diesel, and battery. The best-applied system achieved (COE) of 0.21 $/kWh. Taghavifar and Zomorodian (2021) investigated a micro-hybrid system of solar/wind energy for buildings with the ability to sell back excess electricity. Two cases have been studied using an inflation rate of 10% and wind speed of 6.8 m/s. The solar-wind grid has a net present cost (NPC) of $49,022, a renewable fraction (RF) of 85.5%, and a cost of energy (COE) of $0.0024. Then, the solar-wind-grid gen used a NPC of $224,430, RF of 63.6%, and COE of $0.0272. The results showed that the simple system is suitable for a low inflation rate, while increasing the inflation requires additional equipment. Also, increasing the wind turbine leads to reducing the NPC, and as the wind speed increased, the COE reduced. Only a 5% increase in the inflation rate raises the RF from 14 to 50%.

Sim and Suh (2021) simulated photovoltaic (PV) and ground-source heat pumps (GSHP) systems to maximize the total life cycle cost (LCC) of high energy-consuming buildings. The simulation is implemented various costs and efficiencies by using the EnergyPlus and DesignBuilder software. Then, the results are optimized using a heuristic solution and multi-objective genetic algorithm. The results became guidelines to develop and integrate renewable energy systems for newly built buildings as a decision-making tool, as it could be for existing buildings to achieve better energy efficiency. Pirmohamadi et al. (2021) simulated existing office buildings to decrease energy usage and increase the building’s energy efficiency. The simulation employed a solar thermal system to reduce the building’s CO_2_ emissions by using the DesignBuilder software. Various energy resources are introduced toward the zero-energy building, such as solar energy, electricity, and natural gas. The results concluded that there are fuel consumption and CO_2_ reductions. Solar thermal increased the thermal efficiencies to 47%, which saved about $318 annually. The zero-energy building system could be supported by 24 of 2 kW wind turbines, 3 of 9 kW wind turbines, and 460m^2^ of PV.

Dong et al. (2021) analyzed the operational performance by several factors to achieve a zero-energy building. A case study investigated the reduction of the life cycle carbon emission through 1 year in China. The results showed that the building had a good indoor environmental quality, providing a remarkable point that DesignBuilder proved the minimum area per capita was 42m^2^/person. Furthermore, it revealed guidance for zero-energy buildings design using photovoltaic (PV) panels on the building rooftop. Al-Saadi (2021) applied a multiphase energy audit using the DesignBuilder software on a building’s operation deficiency. Various energy management tools are implemented, such as readjusting the thermostat set point, changing the air conditioning schedule, utilizing energy-efficient lights, and increasing the airtightness. The results proved that reduction of energy consumption and cost is applicable by 56% and 54%, respectively. The post-energy audit phase indicated an energy saving of 34%.

On the other side, wind energy extractors are studied on port building to provide renewable energy. Shun and Ahmed (2008) experimented a wind and solar energy on building using a small hybrid ventilation device. The experiment is done using a wind tunnel testing. The results showed an improvement of the operational performance of the hybrid device compared to the conventional roof top ventilators. Chong et al. (2012) experimented an innovative 3 in 1 wind-solar-rain harvester renewable energy design for urban. Novel power augmentation guide vanes surround a VAWT which is tested inside the wind tunnel. The results showed the augmentation vanes improved the starting behavior and increased the rotational speed of the VAWT by 73.2%. Elkhoury et al. (2015) examined a micro VAWT wind turbine experimentally into a wind tunnel. Then, using a numerical method, the same turbine is investigated by a large eddy simulation (LES) model. Both models are used to investigate the turbine performance affected by the wind speed, turbulence intensity, airfoil shape, and strut mechanism with and without variable pitch. The findings revealed that thicker airfoils more likely have better performance for fixed-pitch VAWT with high solidity ratio. Li et al. (2016) experimented a straight-bladed VAWT that has 2 blades of NACA0021 airfoil to investigate the output power performance. A real case boundary condition is applied to the experiment, while the wind velocity is measured in the wind tunnel to verify the wind velocity in the test field. From measuring the power and torque performances of the VAWT, output results provide a theoretical guiding to the development of VAWT.

Xu et al. (2021) conducted a numerical investigation to examine the wind turbines performances in an array by arranging them between two buildings, in urban areas. Two arrays of wind turbines uniformly arranged in a line, perpendicular to the free stream. The result shows that the asymmetric wake achieves a greater mean power coefficient. The location of the array is significantly affecting the wind, which could be affected by the building and become nonuniform along with the array, which affects the turbine power performance. The wind directions in urban area at 15 degrees achieved the maximum power coefficient. Also, the array in urban areas always has a greater output power than that of a single turbine. Fan et al. (2021) numerically investigated the interactions between turbines, street trees, and buildings in complex urban environment, using a LES model. The results showed that the tall trees extract energy from the mean momentum, while trees lower than the buildings reduce turbulence production. The trees improved power output by 16% and decreased power fluctuation by 5.2%. It is important to take the tree heights into considerations when designing an array of rooftop wind turbines. Dai et al. (2022) simulated the wind flow over flat rooftops of tall buildings with wind angles of 0, 22.5, and 45 degrees. Different roof’s corner modifications are investigated, such as the benchmark, recessed, chamfered, and rounded. Meanwhile, the flow characteristics are studied, such as the velocity, turbulence intensity, skew angle, wind energy, and turbines installation over roofs. Rounded and chamfered roofs had higher velocities than those of the benchmark and recessed roofs. The wind energy increased over the rounded roof, which allows it to be ideal for the installation of turbines.

Di Vaio and Varriale (2018) have reviewed the changing of maritime ports into green energy users. Based on the regulatory frameworks, the effective practices for environmental sustainability are suggested. Also, the managerial accounting instruments for assessing, monitoring, measuring, and controlling the seaport are introduced. Giudice et al. (2022) investigated the literature for the sustainable and innovative development of shipping and seaports. The study investigated the digitalization and new technologies that can help in the creation of sustainable maritime industry. Di Vaio and Varriale (2020) studied the ability of applying digital platforms for business process management. The model is meant to control the operational processes in seaports.

At the same time, Egyptian ports contribute to the Egyptian economy and social development. However, it is highlighted that there is wide research gap about the green development of ports. Few research have applied the green maritime port application; meanwhile, there is no one who has applied the optimum strategy using experimental and real case application with an optimized strategy. Also, there are no existing studies related to the green port application in Mediterranean port, as well as in Alexandria port, Egypt.

**Fig. 1:**
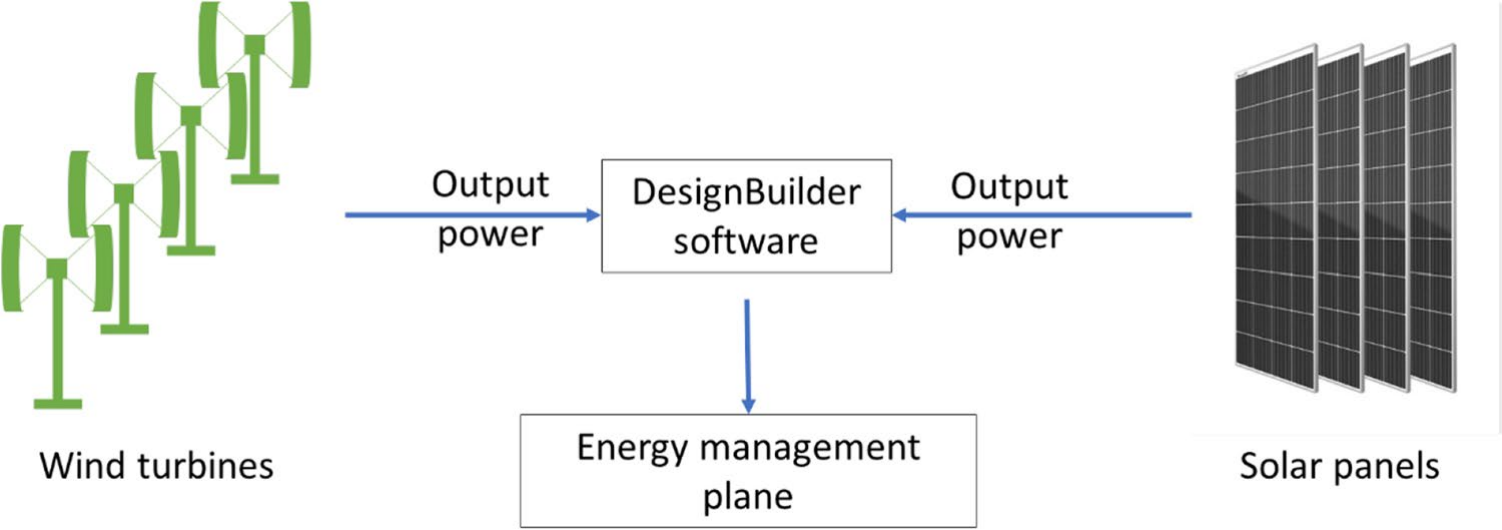
Conceptualization of the study

Therefore, the contribution of this work is to evaluate an optimum strategy through an optimization of an energy management system for a green port in Egypt that reduces the port’s energy consumption by reducing the port’s building consumptions. Renewable energy as the solar energy and wind energy is used with the coupling optimization method to specify optimum strategy (see Fig. 1). This study aims to integrate the best application at port using experimental and numerical approaches. Onsite solar arrays are applied to address their measures regarding the combination of green development strategies for adopting a low-carbon port model by harnessing renewable energy. The DesignModeler software simulates solar energy with the capacity of the solar arrays and validated the real model. Also, the ANSYS Fluent software simulates wind energy by using wind turbines on port’s buildings. Therefore, the objectives of designing a green maritime port are summarized as follows:

- Investigate the solar and wind conditions using an installed onsite weather station.
- Impalement the solar energy arrays onsite inside the port.
- Perform a simulation to determine the solar energy using the DesignBuilder software, and validate the numerical model with the onsite model.
- Simulate the wind energy using the ANSYS Fluent software.
- Perform a performance analysis on both systems with Pareto optimality analysis.

## Numerical methodology

**Fig. 2:**
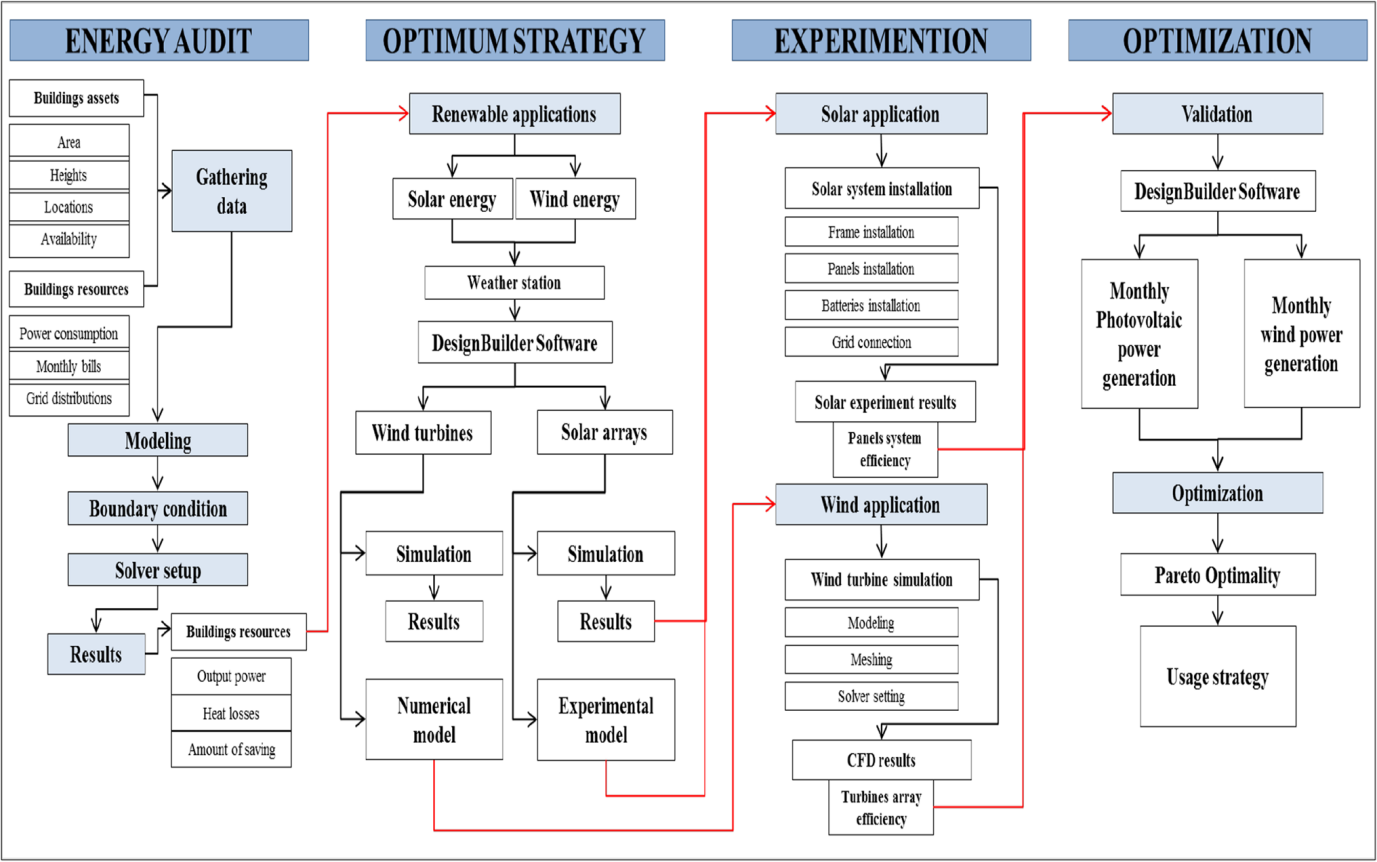
Flowchart of the used methodology

The numerical methodology applied for this study takes four main steps for the auditing of the port’s buildings. Applying renewable energy such as the solar and wind is the leading strategy. So, the first step is to take a wide overview about the available resources, while auditing all the building resources and consumption during the whole year. Then, a numerical analysis is conducted to measure the required solar and wind applications using the DesignBuilder software. Gathering the output results from the DesignBuilder allows to implement a real case of the solar system and simulate computational fluid dynamics for the wind system. The actual results are optimized together to reveal the optimum wind and solar applications for each building, which achieved overall integrity. All these steps are summarized in Fig. 2.

The interplay between solar energy and wind energy is the best way to embark on green energy within the MINDTAP port. So, each one has been studied separately and evaluated to be merged with the system. The available buildings will be evaluated to provide the actual surface area that will allow the installation of solar and wind energy extractors. Total facilities are listed in Table 1, which gives insight how the arrangements will be. There are 6 buildings available. Those would be applicable to be used for the current project based on the solar panels’ areas and the turbines’ surface areas.

**Table 1:**
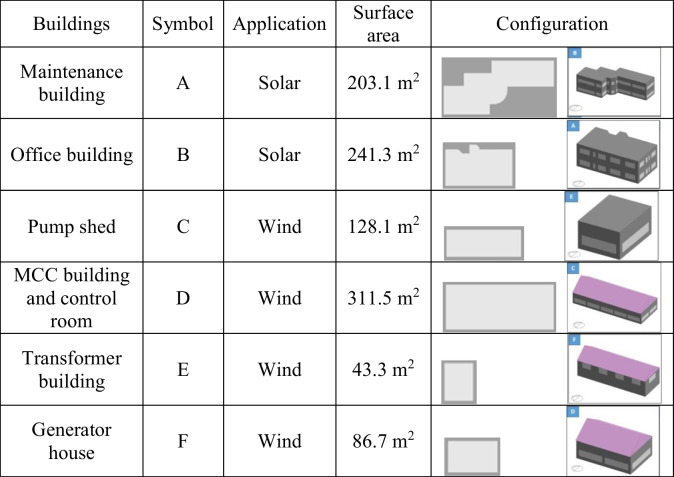
Available building floor areas for the project

According to the roof’s topology, there are three buildings with inclination, which would not be suitable to support the installations for solar panels, even the sun orientation. As well, the office building also is sufficient for wind energy. So, solar energy will be installed on the building A and B, whereas buildings C, D, E, and F are service buildings used for the wind turbines.

### Solar energy

#### Design of the solar system

Maritime ports are one with the highest power consumption. These ports are demanding more power yearly due to the rapid growth of world’s trade. Here in Egypt, it is almost the same as any port worldwide, especially in the port of Alexandria. MIDTAP port is a main gate of the energy transportation by providing efficient operations. However, this high amount of power consumption is producing a tremendous amount of pollutants. Therefore, going for green energy is the best way to overcome such problems. It will provide green generated power; hence, it will cut the pollutants.

There are many sources of green energy; however, the solar energy is the most popular one and available most of the year, especially during the summer months. Designing a solar system for is challenging and required a good study to ensure the accurate installation of the system. The DesignBuilder software is a perfect design and analysis tool for solar systems. Also, it would be very useful in analyzing the output of the system during the operation and recorrecting the results for an energy audit.

The energy management plan is set for the solar system over the year of 2021/2022 to be implemented on buildings A and B. But first, a required amount of energy is calculated by the software to be installed on the roof of buildings A and B. This amount is based on the available energy provided from the sun source.

#### Experiment of the solar system

##### System preparation

**Fig. 3:**
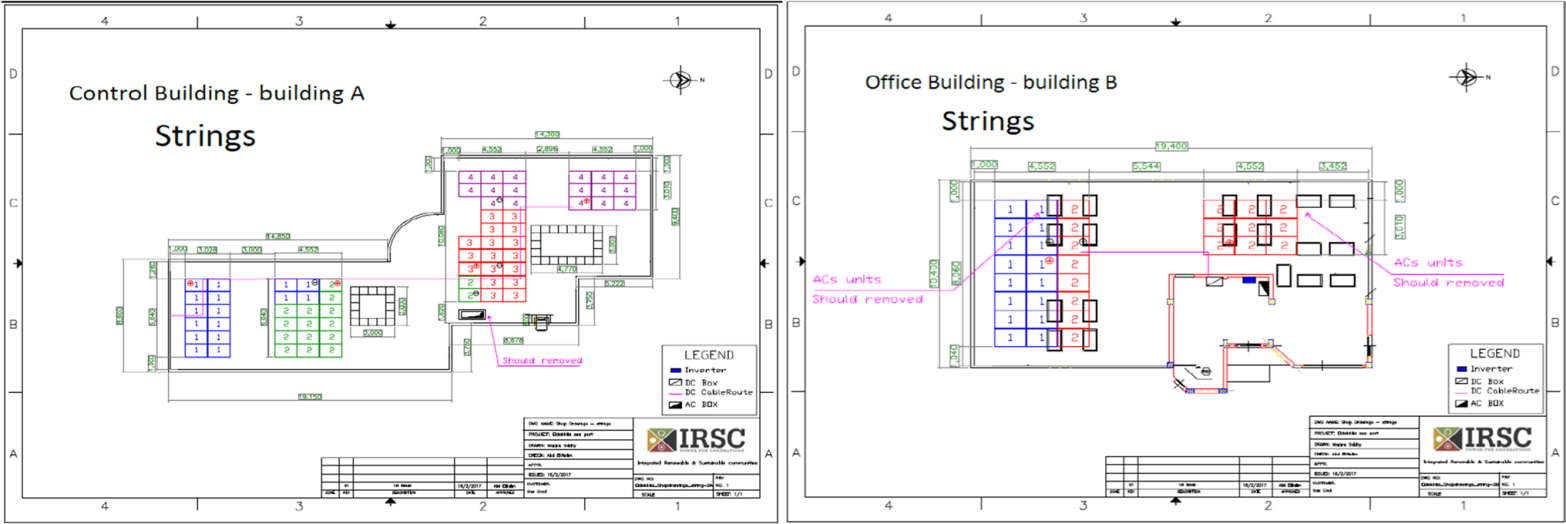
Strings of the control room building (A) on the left and the office building (B) on the right

**Fig. 4:**
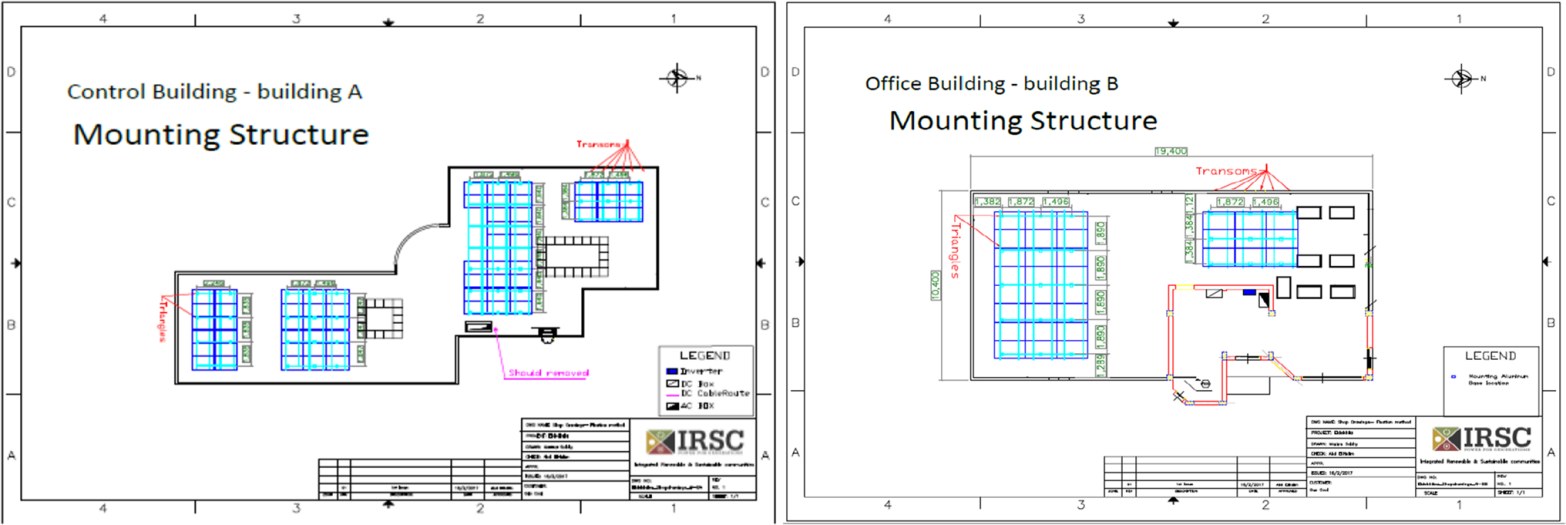
Mounting structure of the control room building (A) on the left and the office building (B) on the right

The design and implementation of the solar system are carried out for both buildings. Basic system’s drawings are used as showing below for the control room building (A) and the office building (B). That implementation was based on the design of strings (see Fig. 3) and mounting structure (see Fig. 4), to show the fixation location.

##### System implementation

**Fig. 5:**
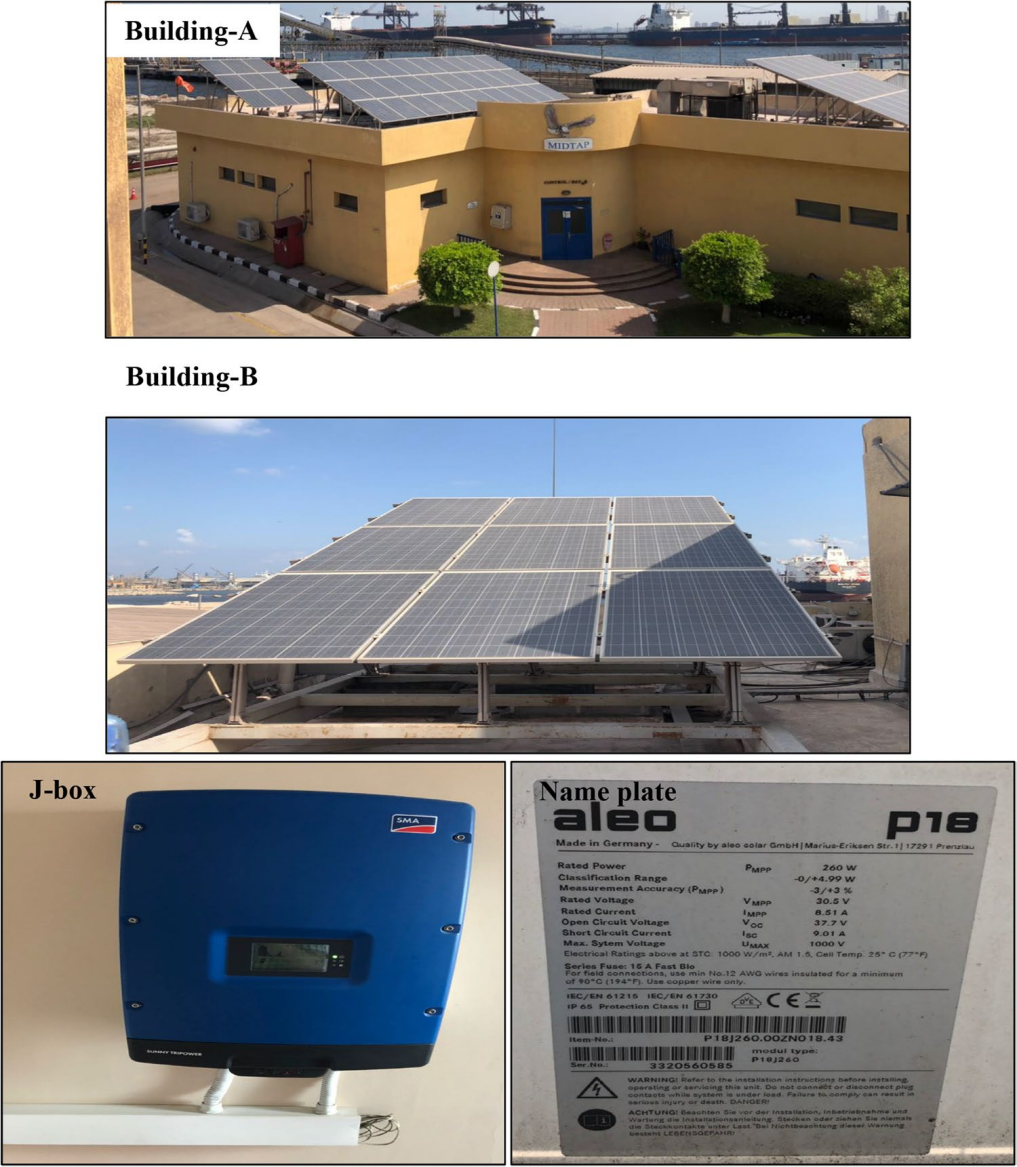
Solar panel installation on building A and building B. On the bottom, the junction and control box of the system and name plate of the installed panel

The outputs from the DesignBuilder software are implemented into the integrated solar system. The system consists of panels. Building A consists of 9 panels, while building B of 3 groups has 9, 30, and 18 panels, respectively (see Fig. 5). The panels are set on a steel frame and angled by 25 degrees. Then, the panels are wired together and connected to the main source of the system.

**Fig. 6:**
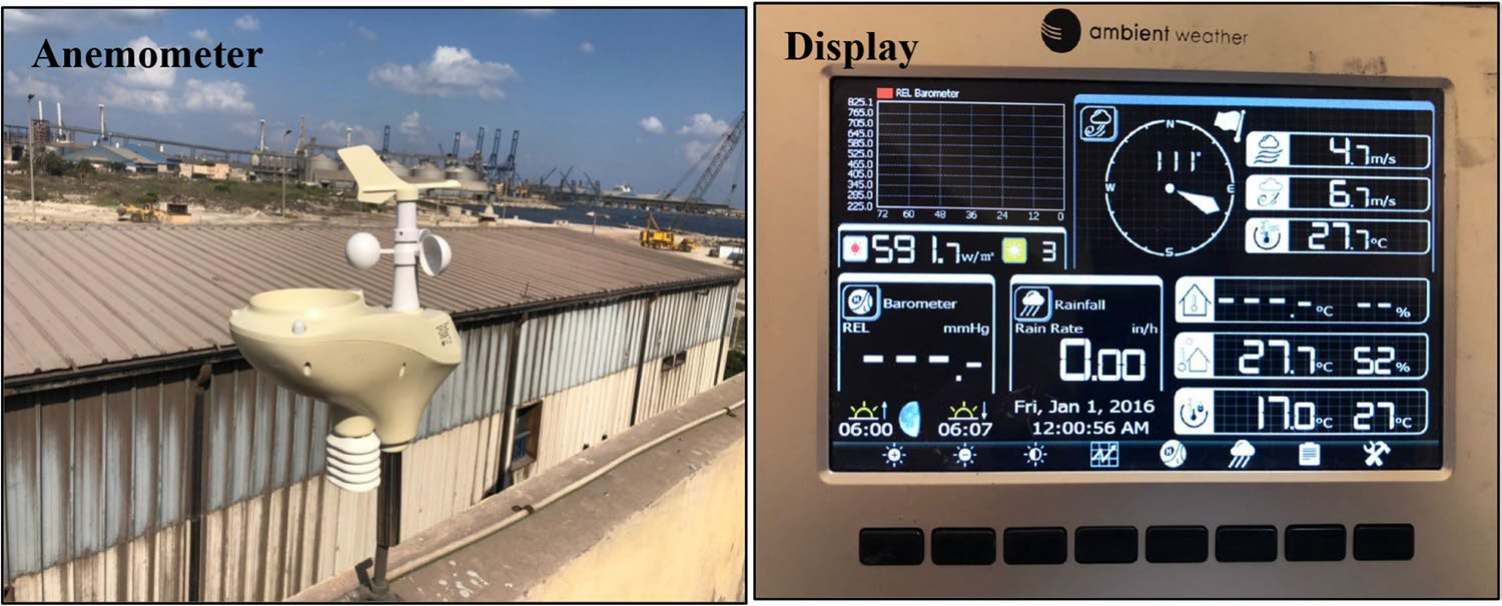
Weather station for observing the local weather condition installed on building B which consists of an anemometer and a display

Meanwhile, a weather station is installed to gather a real hourly data of the wind and solar systems. The station observes the local weather condition by using anemometer, display and junction, and control box (see Fig. 6).

### Wind energy

#### Design of the wind system

High wind energy intensity is available at maritime ports when there are no obstacles to disturb the upstream wind velocity. Therefore, harnessing this energy is promising to provide green energy. Preliminary wind energy system is studied based on the available data on location. The DesignBuilder software is used for the process, which is a vital tool that analyzes the energy systems, used for the solar system. The DesignBuilder has powerful capabilities to study the energy plan over the year under different operation conditions. Different types and sizes of wind turbines are investigated, and the small to medium sized vertical axis wind turbines (VAWT) are the best choice. However, the number of turbines is specified based on the available surfaces of each building of buildings C, D, E, and F.

The energy management plan is investigated also for the wind turbines though 2021–2022. This plan is to provide improvement of the wind turbine operations though the year. The plan also is to allow the integration of the wind turbine with the solar planes at the same time. The strategy is to ensure the sustainability of the green power production and the smoothing of the output power without any faulty procedures. The port’s power consumptions over the year is gathered from the monthly paid bills. This allows using the actual data and enhancing the aim of reducing these bills and the conventional power consumptions.

The energy management provides good characteristics that would be used later for applying the wind turbines in the real system. However, the application of the wind turbines on the roof of the four buildings was challenging in the meantime, because of the building structures that need separate structural analysis to avoid any incident, failure, and casualties. Therefore, an alternating method is used, which is the numerical method. A numerical method is used for the remaining analysis and validated against experimental study. So, the output results would be valuable to be reentered to the DesignBuilder software for completing the energy management study.

#### Numerical analysis of wind turbines

As mentioned before, an efficient energy management plan is required to enhance the maritime port of MIDTAP. So, according to the wind energy, good design wind turbine would increase the power captured. More likely, the design of the turbine begins with investigating a single rotor to understand its aerodynamic behaviors, while ensuring good characteristics. A numerical analysis has been used to evaluate the wind turbine characteristics, such as the power coefficient and the arrangement of several turbines. The power coefficient could be used later in the DesignBuilder software for the energy management strategy. However, the layout of the turbine on the buildings seems to be complex, according to the limited areas of the rooftops.

The computational fluid dynamic (CFD) method is used for applied turbines to study the influence and the interaction of the turbine aerodynamic on the efficiency improvement of the complete system. At first, a fluid domain is established based on the experiment that done by Elkhoury et al. (2015). This simulation used a 3D modeling of the turbine by the ANSYS Fluent software V18.0 to domain the rotor, at the AAST labs.

##### Numerical model

**Fig. 7:**
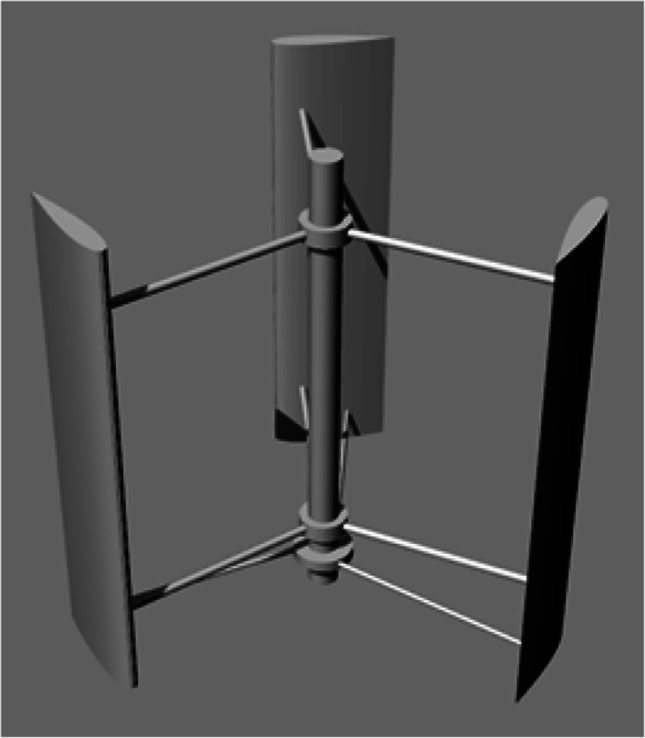
H-Darrieus VAWT rotors using NACA0018 with fixed pitch angle

H-Darrieus VAWT is used to sustain a continuous high output power of the wind energy. The blade’s airfoil is the NACA0018 with a fixed pitch angle setup, and the blade’s angle of attack was set to be zero degree. The Darrieus turbine was selected to be investigated at the Alexandria port, which is suitable for the port’s boundary conditions to interact with the wind velocity. The overall Darrieus rotor diameter is 0.8 m, while the rotors has 0.8-m height (see Fig. 7).

**Fig. 8:**
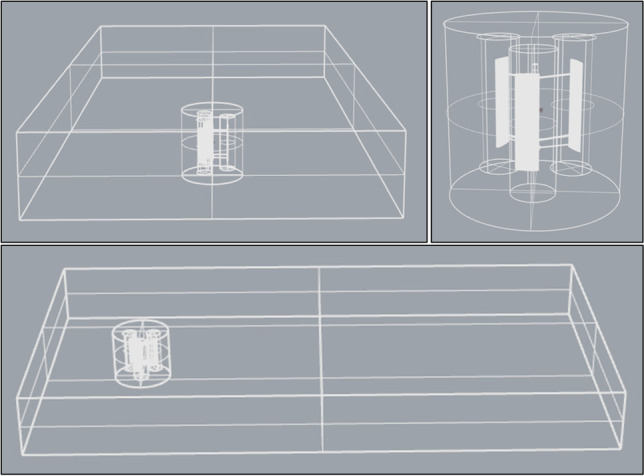
The configuration of the fluid domain employing the turbine

The Darrieus rotor has 3 fixed blades, rotating by angular velocity ranging between 0.25 and 1.5 rad/s. The turbine rotates counterclockwise, with a wind density of 1.25 kg/m^3^. The fluid domain has boundaries length, width, and depth of 16D, 11D, and 2.25D, respectively. Then, the main domain is divided into sub-domains to improve the mesh of the domain. Thus, the rotor is placed inside a blade domain that has a diameter of 1600 mm and length of 1500 mm, which is in the middle between the top and bottom boundaries. The rotor domain is placed 3 times the rotor diameter of the free stream inlet. Each blade enclosed inside a cylindrical enclosure has a diameter of 400 mm and length of 1200 mm. The three cylinder enclosures are showing in Fig. 8.

The boundary condition setup is used with a velocity of 8 m/s, for the air inlet boundary side. The air outlet from the domain under atmospheric pressure is from the pressure output side. Both sides and the top and bottom walls are considered symmetry. All walls, such as the blades, rods, and shafts, are set with no-slip boundary condition. Four interfaces are assigned relative to the contact surfaces between the main domain with the rotor domain and the rotor domain with each of the three blade domains.

The sliding mesh motion (SMM) method is used to rotate the rotor domain as well as the three blade domains. Six rotational velocities are used based on the tip speed ratios (TSRs) of 0.25, 0.5, 0.75, 1.0, 1.25, and 1.5, to simulate different cases. Time step is selected based on each TSR as the minimum value (∆*t*) (see Table 3). The minimum time steps were evaluated to reduce the simulation time, while maintaining accurate solutions.

The Darrieus turbine is modeled based on the given reference (Elkhoury et al. 2015) as shown in Fig. 7. The following table (see Table 2) shows all the necessary parameters that could be used for modeling the turbine rotor. Each blade is centered inside the blade domain by 0.25c of its chord; at this point, the blade is connected to the main shaft by the main link.

**Table 2:** The H-Darrieus turbine parameters (Elkhoury et al. 2015)

Parameter	Value
Diameter	800 mm
Height (blade span)	800 mm
Chord	200 mm
Mechanism	Fixed-pitch angle
Number of blades	3
Material of blades	Aluminum
Axis of rotation	Z axis

The model solver was used in this study which is a double precision solver, for the unsteady implicit coupled pressure based. A LES simulation with variable Smagorinsky coefficient is applied to the model having a bounded central difference discretization scheme. The discretization scheme ran with second-order upwind-based, while the cell-face pressures ran with the second-order interpolation scheme. A minimum of 20 iterations were necessary for the model convergence, when the convergence criterion is set 1E-6 of residuals.

##### Verification and validation

Validation against an experimental and numerical models (Elkhoury et al. 2015) is necessary to assure the accuracy of the results.

##### Model verification

**Fig. 9:**
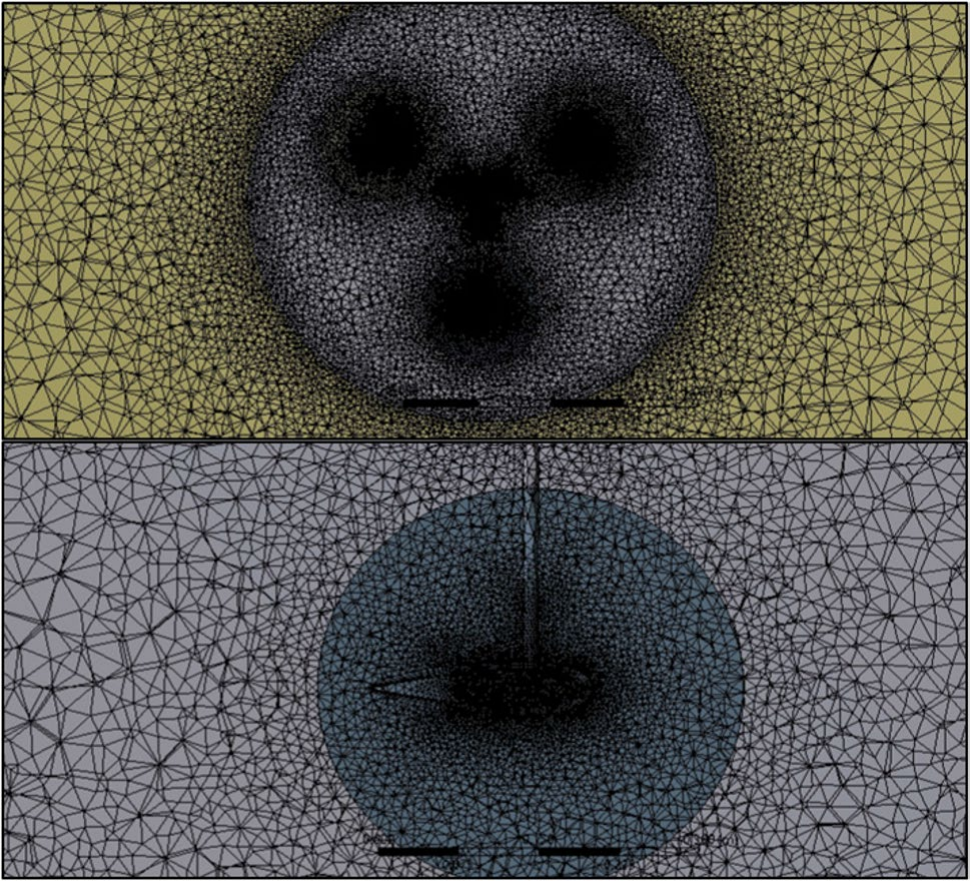
Grid of the turbine’s rotor and blades on the top and bottom, respectively

**Fig. 10:**
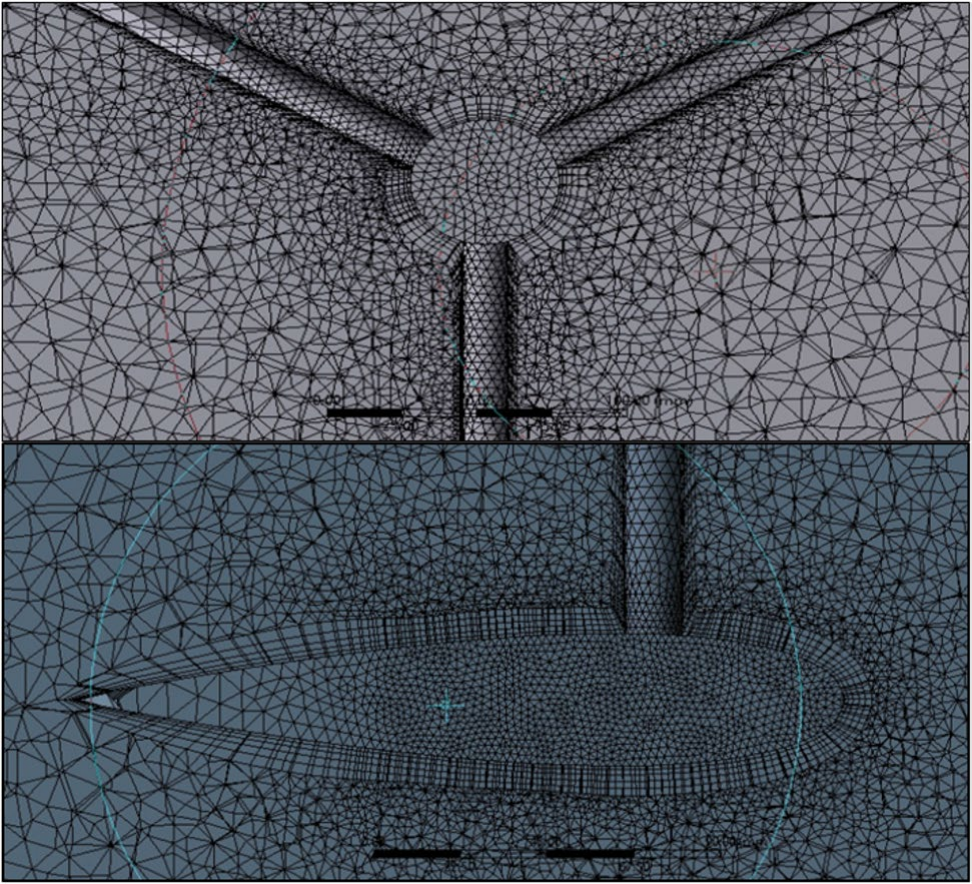
Mesh inflation around the connecting links and the blade on the top and bottom, respectively

Unstructured mesh is used for the model to run the SMM technique. The domain is divided into mesh using a fine mesh with a minimum and maximum cell size of 1.5 mm and 500 mm, respectively. Fine sizing functions are used for each of the edges and faces of the blade, domains’ interfaces, connecting links’ surfaces, and the shaft’s surfaces, to handle the mesh quality. A geometric expansion ratio of 1.2 was employed to accurately resolve near-wall flow structures. Each of the rotating domains had finer mesh for its body of 1.5 mm for the rotor’s domain and 1.5 mm for the blades’ domains (see Fig. 9). Inflations to enhance the mesh quality of 5 layers are set around each of the blades, the connecting links, and the shaft, with a minimum cell sizing of 1.5 mm (see Fig. 10). The final mesh element number is 13.27 million elements, which is recommended based on the given reference (Elkhoury et al. 2015), as also revealed from the mesh sensitivity analysis.

Grid study is carried out on three different mesh resolutions, the coarse mesh, the medium mesh, and the fine mesh, in order to select the proper mesh. The recommended mesh elements were ranging from 5 to 15 million. The mesh element numbers of the three cases are compared to the calculated power coefficient to determine the optimal mesh number. In case number 1, the mesh element number is 7.37 million. In case number 2, the mesh element number is 13.27 million, and the power coefficient error is 38.4%. In case number 3, the mesh element number is 15.4 million, and the power coefficient error is 4.42%. So, the value of 13.27 million elements is selected as it achieved a good characteristic, the same with the 15.4 million elements.

##### Model validation

**Fig. 11:**
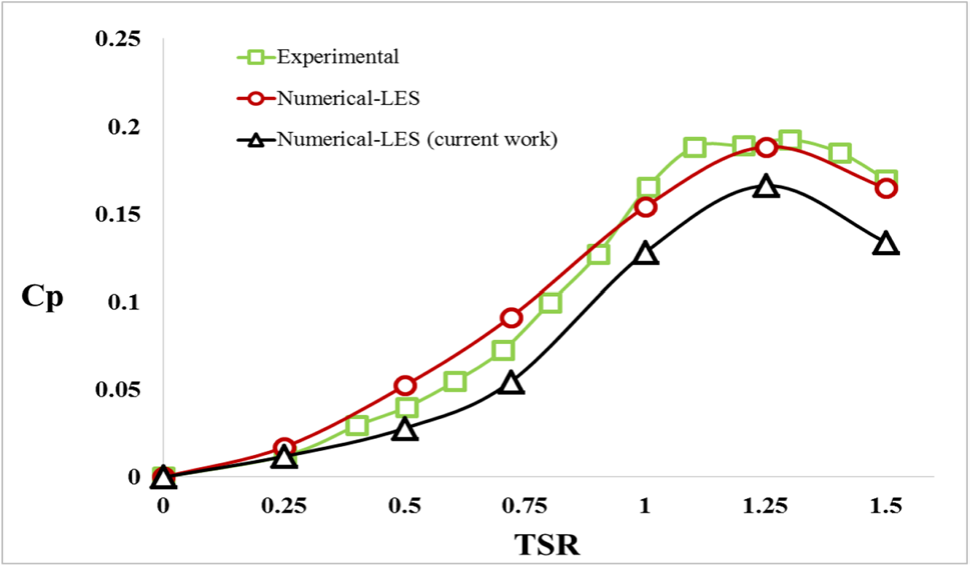
LES validation against an experimental model and a numerical model (Elkhoury et al. 2015)

The LES model has been investigated; this model was recommended by Elkhoury et al. (2015). The six tip speed ratios 0.25, 0.5, 0.78, 1, 1.25, and 1.5 were used as the numerical data were provided (see Table 3). Power coefficients are measured and studied to investigate the turbine performances. All the result values are averaged of 2 full rotations that are achieved by 72 steps, after the model became stable. These values were compared to the experimental and the numerical data as in (Elkhoury et al. 2015) (see Fig. 11). The model had an average error of 2%, so the LES is selected. Hence, the maximum power coefficient is achieved of 0.082, when the tip speed ration is 0.98 (see Fig. 11).

**Table 3:** Tip speed ratio related to the angular velocity

TSR	w (rad/s)	∆*t*	∆t-10 degrees	*C*_*p*_
0.25	5	0.0035	0.0349	0.012
0.5	10	0.0017	0.0175	0.028
0.75	15	0.0012	0.0116	0.054
1	20	0.0009	0.0087	0.128
1.25	25	0.0007	0.0070	0.166
1.5	30	0.0006	0.0058	0.134

##### Turbines arrays

The arrays of vertical axis wind turbines are examined on four buildings in the port of MIDTAP urban environment. The vertical axis wind turbine performances are investigated using the LES model. The local wind speed is taken into consideration; however, its mean values averaged between the onshore and offshore wind speeds, which is 3.75 m/s.

**Fig. 12:**
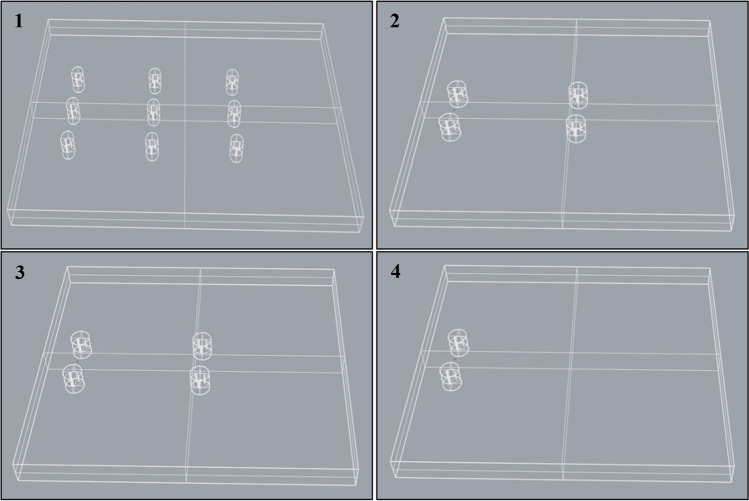
Arrays of VAWTs, arranged 3 × 3 for building C in domain 1, 2 × 2 for building D in domain 2, 2 × 2 for building E in domain 3, and 2 × 1 for building F in domain 4

Four buildings are usable for wind turbines, building C, D, E, and F. The available active areas of the buildings’ roof would accommodate 9, 4, 2, and 4 VAWTs on roof top, respectively. Four arrays of wind turbines are uniformly arranged in a line, perpendicular to the free stream. The location of each wind turbine along with the array is significantly affecting the output power. Distances between VAWTs are used based on previous numerical studies (Fan et al. 2021; Xu et al. 2021). Spacing between turbines are 3 times the diameter perpendicular to the wind direction and 10 times the diameter along the wind direction (Xu et al. 2021) (see Fig. 12).

The roof areas of building D and building E are relatively close; therefore, both buildings have the same 4 turbines and same layout. So, only building D is simulated to reduce the simulation time.

### Energy audit and optimization

Nevertheless, regarding to the methodology chart, the final step is to optimize the results for operation and the power generation (see Fig. 2). Optimizing various results from power generation that were resulted by the solar system and wind turbines could not be done manually efficiently. The gathered data from the solar system and the weather station are reanalyzed and optimized for energy management plan. These data show the actual weather condition affecting the output power of the solar system. The DesignBuilder software is used to simulate the results of the giving data according to the real applied condition. The green power production is investigated for every month over the year supporting for sustainable strategy. Power consumption bills of the port provide a scheme of the used power, which is used at the simulation to achieve the strategy of reducing these bills. Also, it is used another time to reanalysis and evaluates the output results of the numerical simulations of each building with respect to the wind energy. Then, the results from both solar and wind analyses are optimized used the Pareto optimality method for evaluating the optimum power generation and power consumption.

## Results and discussions

### Solar energy

#### Theoretical and practical implications

Simulating the solar energy system using the DesignModeler revealed very interesting results compared to the actual data set. The following report shows a summary of the simulated parameters for the grid-connected system at the MIDTAP port.

Control building A consists of two sub-arrays, as shown in the following report. The first one has 17 modules in parallel 2 strings of the total 34 units with a nominal power of 260 Wp and an array global power nominal of 8.84 kWp at an operating condition of 7.88 kWp (50 °C), with 464 V and 17 A. The second sub-array has 16 modules in parallel 2 strings of the total 32 units with a nominal power of 260 Wp and an array global power nominal of 8.32 kWp at an operating condition of 7.41 kWp (50 °C), with 437 V and 17 A. Meanwhile, the total array global power nominal is 17 kWp, total 66 modules, with a covering area of 108 m^2^ for the whole module and cell area of 96.4 m2.
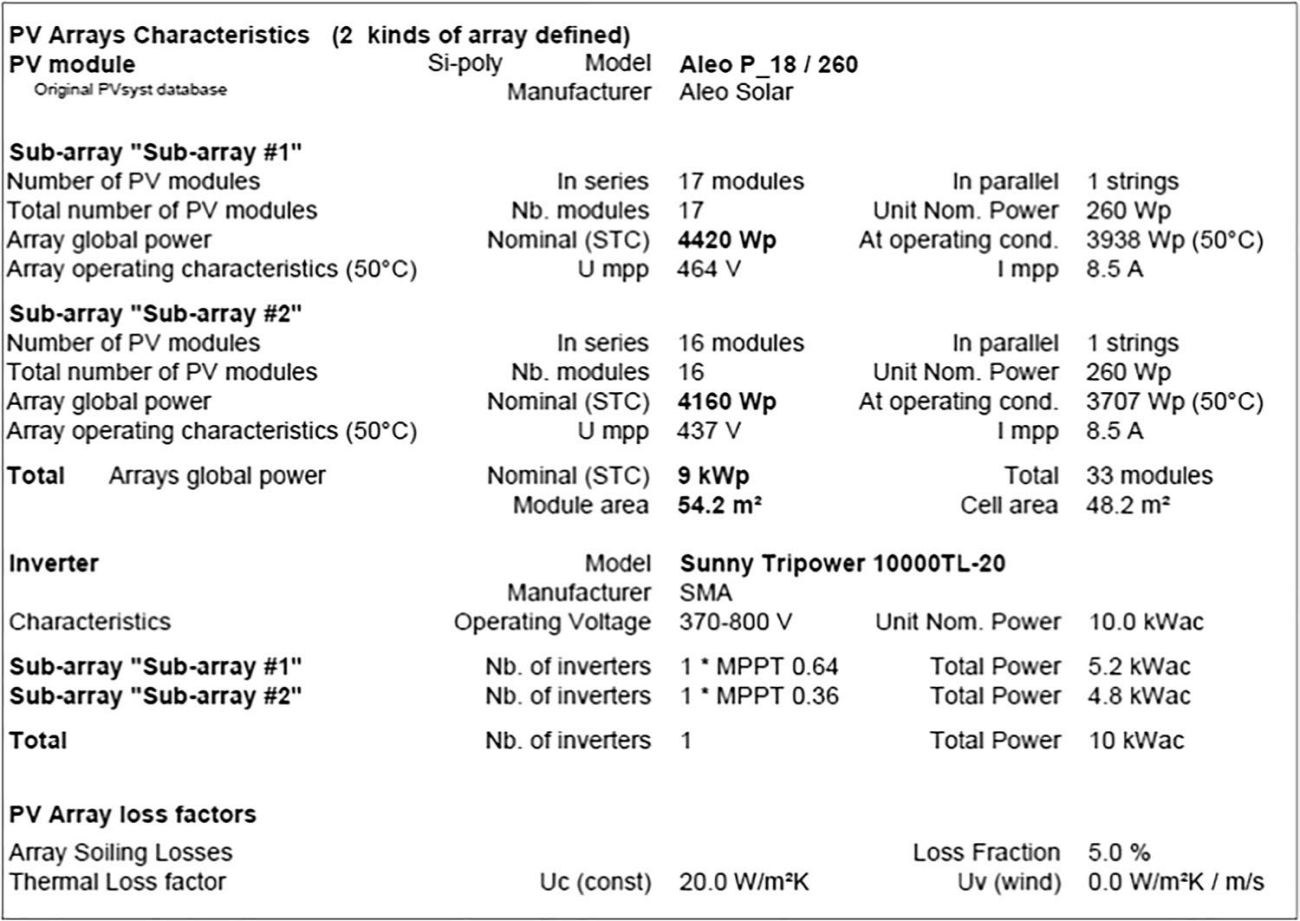


On the other side, the office building B also consists of two sub-arrays. The first sub-array has 17 modules in parallel 1 string of the total 17 units with a nominal power of 260 Wp and an array global power nominal of 4.42 kWp at an operating condition of 3.938 kWp (50 °C), with 464 V and 8.5 A. The second sub-array has 16 modules in parallel 1 string of total 16 units with a nominal power of 260 Wp and an array global power nominal of 4.16 kWp at an operating condition of 3.707 kWp (50 °C), with 437 V and 8.5 A. Both sub-arrays provided a total global power nominal of 9 kWp, total 33 modules, covering area of 54.2 m^2^ for the whole module, and cell area of 48.2 m2, as shown in the following report.
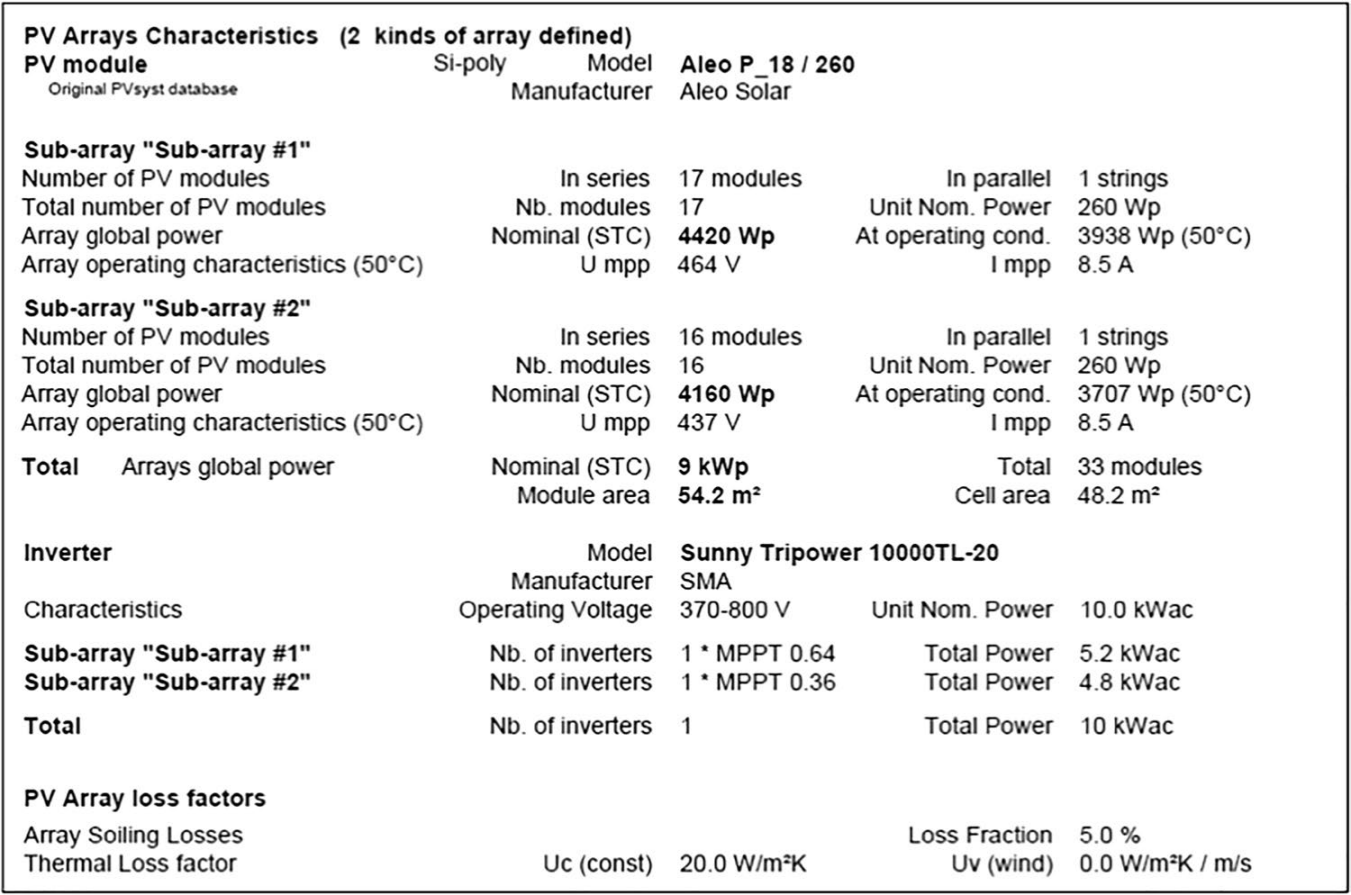


The main system parameters for the control room building A include a PV array of 66 modules, with a total normal power of 17.16 KWp. The array is tilted by 25 degrees, of 0 degree azimuth. Each PV module has a normal power of 260 Wp; its model is the Aleo P_18/260. The generated current then inverted using the Sunny Tripower 20000TL-30 inverter of 20.00 KW ac. Meanwhile, the main system parameters for the office building building B are same with those of the control room building A, but the PV array has 33 modules, with a total normal power of 8.58 KWp. The generated current then inverted using the Sunny Tripower 10000TL-20 inverter of 10.00 KW ac.

**Fig. 13:**
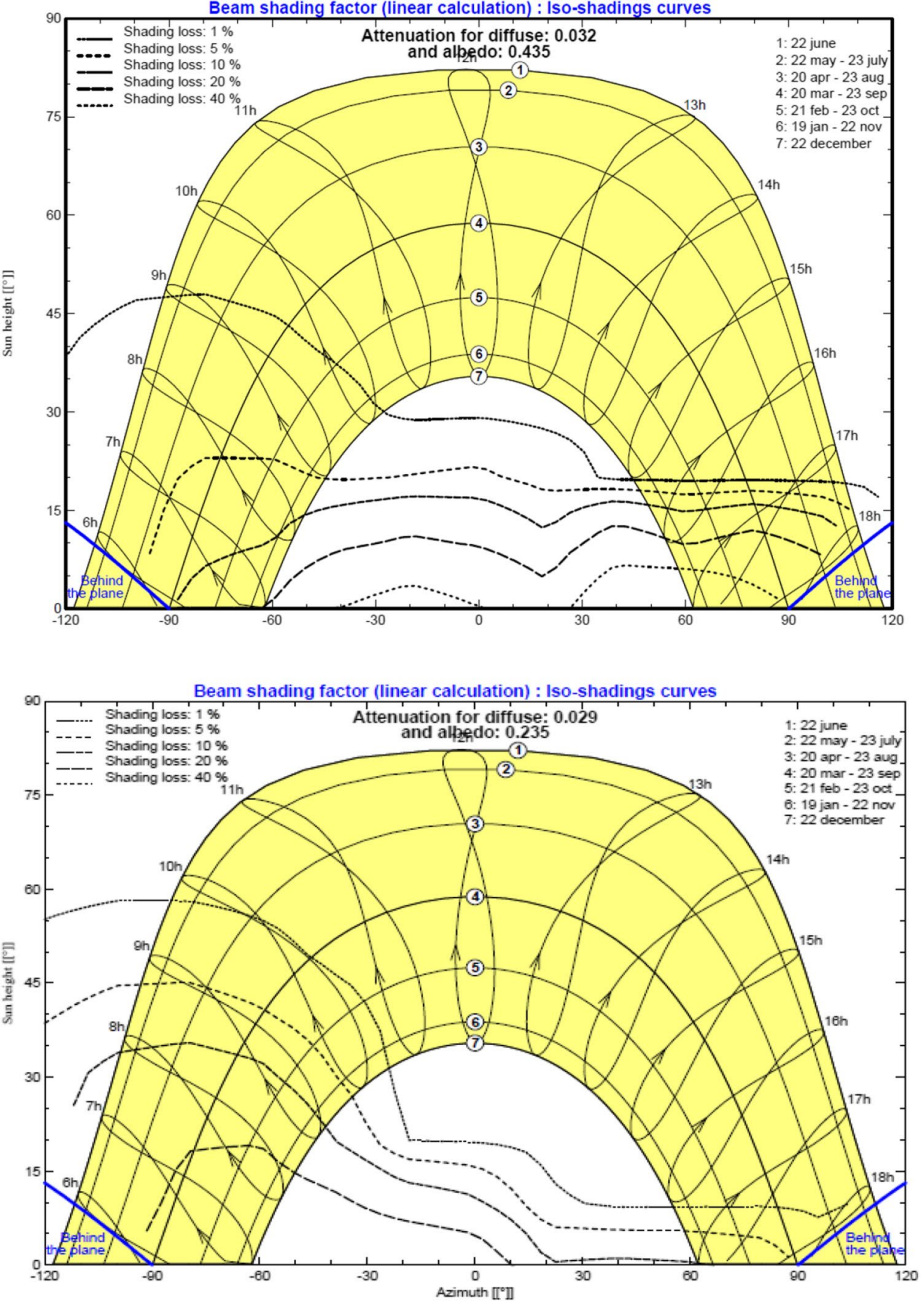
Iso-shadings diagram of the control room building A on the top and the office building B on the bottom

In addition, the near shading definition for the grid-connected system at the MIDTAP port is simulated also (see Fig. 13) in both the control room building A and the office building B.

**Fig. 14:**
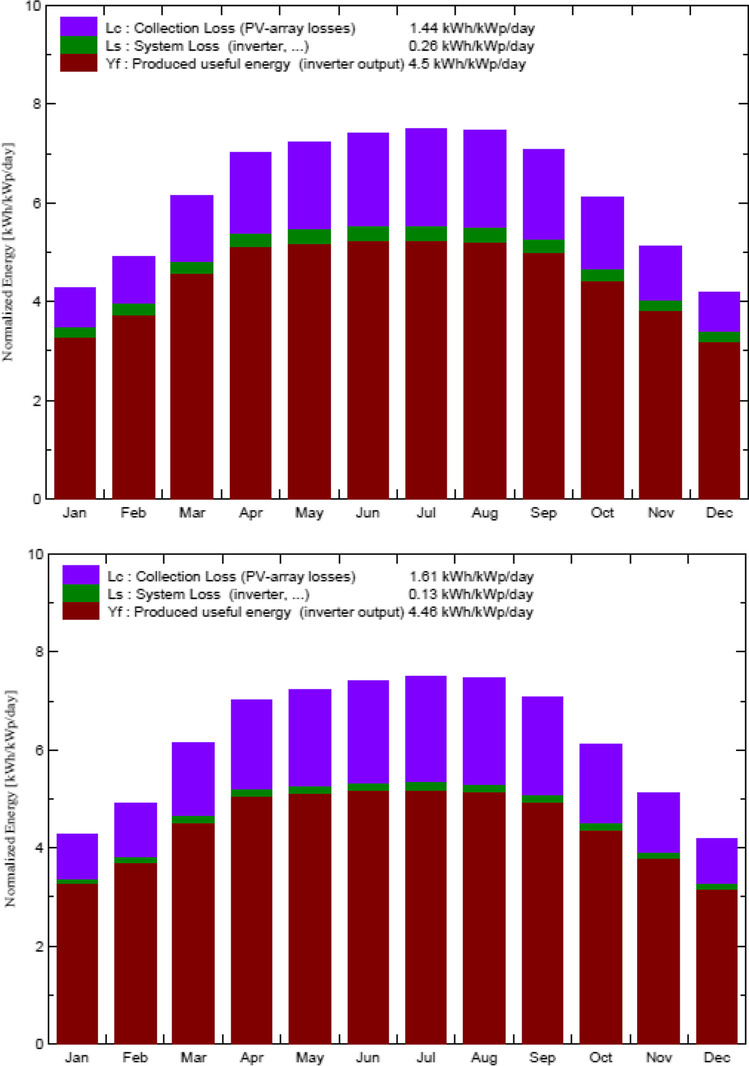
Normalized power productions of 17.16 kWp and 8.58 kWp for the control room building A on the top and the office building B on the bottom, respectively

**Fig. 15:**
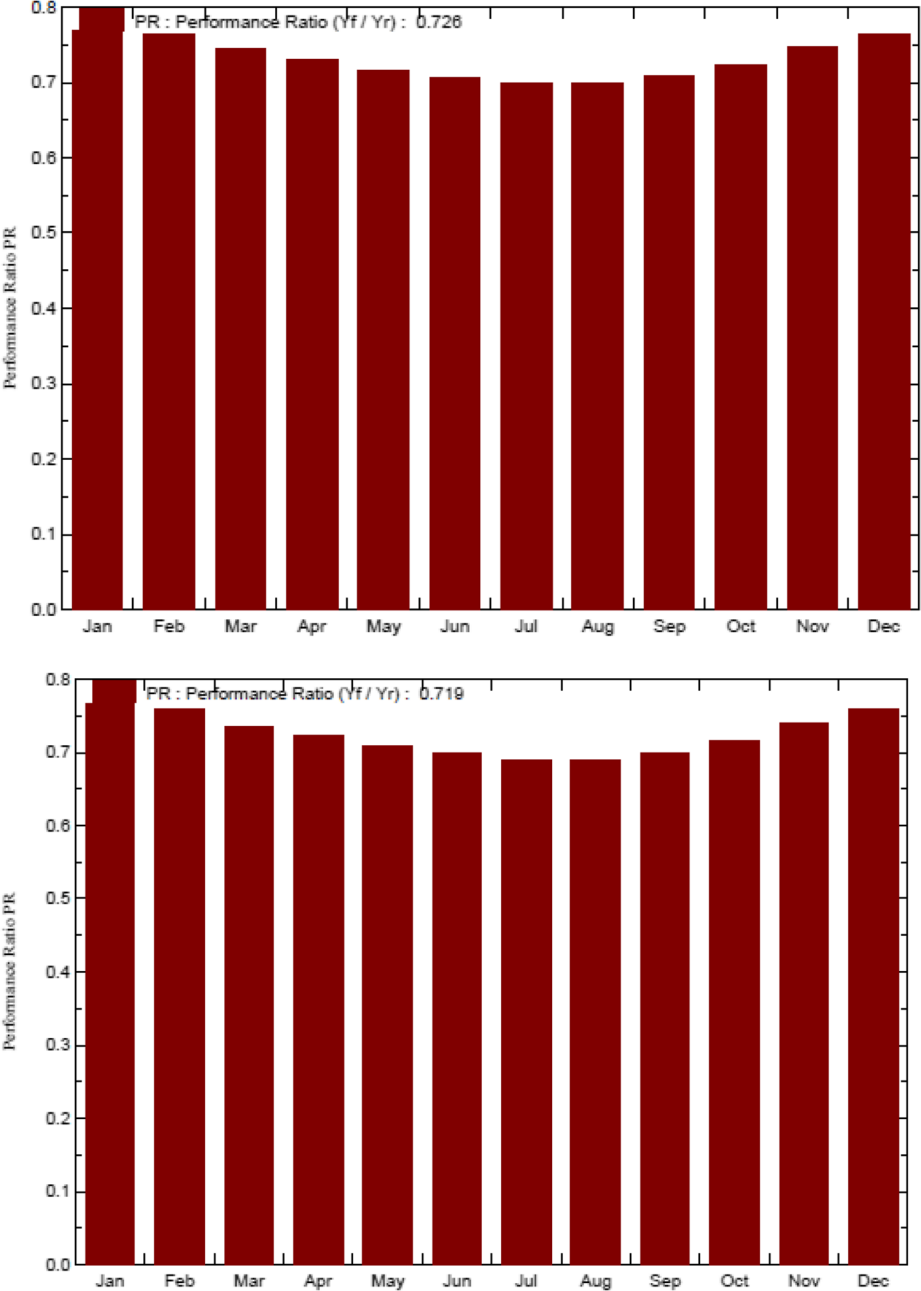
Performance ratio of the control room building A on the top and the office building B on the bottom

The main simulation results for the grid-connected system of the MIDTAP port indicated that the control building A showed an energy production of 28.22 MW/year (see Fig. 14), a specific production of 1644 kWh/kWp/year, and an average performance ratio of 72.6% (see Fig. 15), whereas the office building B had an energy production of 13.97 MW/year (see Fig. 14), a specific production of 1628 kWh/kWp/year, and an average performance ratio of 71.9% (see Fig. 15 and Table 4).

**Table 4:** Balances and main results of the control room building A on the top and the office building B on the bottom

	GlobHor kWh/m^2^	T Amb °C	GlobInc kWh/m^2^	GlobEff kWh/m^2^	EArray MWh	E Grid MWh	EffArrR %	EffS ysR %
January	96.0	12.72	132.3	120.4	1.858	1.745	12.95	12.16
February	108.1	13.33	137.2	124.8	1.907	1.798	12.82	12.07
March	164.4	16.51	190.7	173.9	2.572	2.433	12.43	11.76
April	199.4	19.29	210.4	191.6	2.784	2.837	12.20	11.55
May	231.4	22.85	2240	203.8	2.909	2.752	11.98	11.33
June	239.0	25.55	221.9	202.0	2.850	2.692	11.84	11.18
July	247.0	27.76	232.8	212.3	2.952	2.791	11.69	11.05
August	227.7	27.85	231.2	211.2	2.928	2.774	11.67	11.06
September	188.6	25.59	212.0	194.1	2.719	2.576	11.83	11.20
October	151.0	22.79	189.6	173.7	2.487	2.355	12.09	11.45
November	110.1	18.54	153.8	140.7	2.085	L970	12.50	11.81
December	90.0	14.45	129.5	118.2	1.804	1.695	12.84	12.07
Year	2052.8	20.65	2265.4	2066.7	29.853	28.215	12.15	11.48
	GlobHor kWh/m^2^	T Amb °C	GlobInc kWh/m^2^	GlobEff kWh/m^2^	EArray kWh	E Grid kWh	EffArrR %	EffSysR %
January	96.0	12.72	132.3	120.7	895	870	1248	12.13
February	108.1	13.33	137.2	125.2	919	894	12.36	12.02
March	164.4	16.51	190.7	174.4	1240	1204	11.99	11.65
April	199.4	19.29	210.4	192.1	1342	1304	11.76	11.43
May	231.4	22.85	224.0	204.4	1403	1361	11.55	11.21
June	239.0	25.55	221.9	202.5	1374	1331	11.42	11.06
July	247.0	27.76	232.8	212.9	1424	1379	11.27	10.92
August	227.7	27.85	231.2	211.8	1412	1370	1126	10.92
September	188.6	25.59	212.0	194.5	1311	1272	11.40	11.06
October	151.0	22.79	189.6	174.1	1199	1164	11.66	11.33
November	110.1	18.54	153.8	141.0	1005	977	12.05	1111
December	90.0	14.45	129.5	118.5	859	844	12.38	12.02
Year	2052.8	20.65	22,654	2072.2	14,393	13,971	11.72	11.37

*GlobHor* Horizontal global irradiation, *EArray* Effective energy at the output of the array, *T Amb* Ambient Temperature, *E_Grid* Energy injected into grid, *GlobInc* Global incident in coll. plane, *EffArrR* Effic. Eout array / rough area, *GlobEff* Effective Global, con, for IAM and shadings, *EffSysR* Effic. Eout system / rough area

**Fig. 16:**
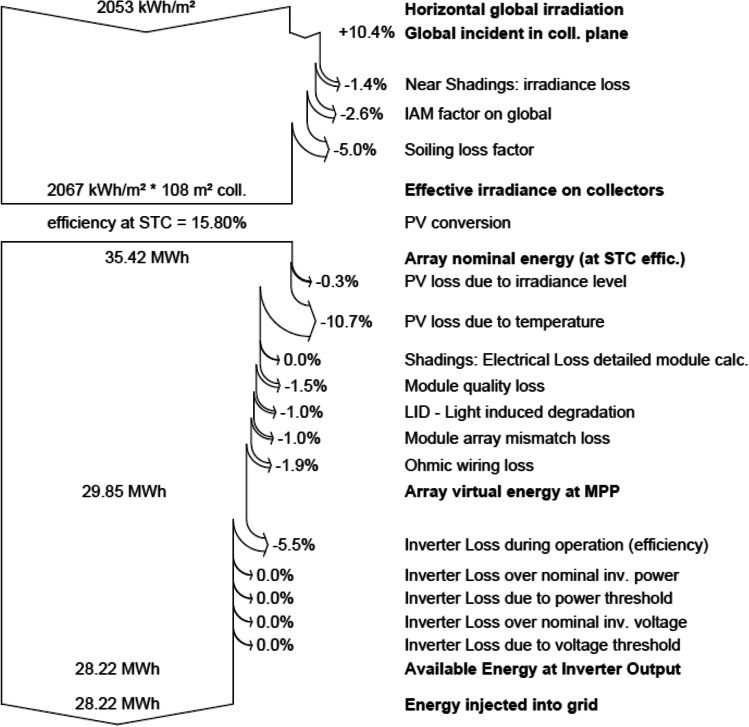
Loss diagram over the whole year of the control room building-A

**Fig. 17:**
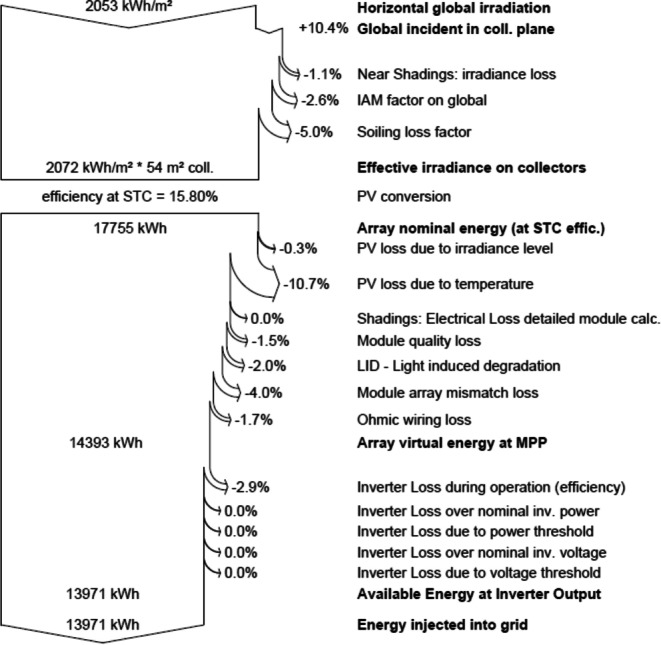
Loss diagram over the whole year of the office building B

Also, the losses diagrams of the grid-connected system are shown the power production, consumption and the losses, which is a good indication of their minimization. Losses of the control room building A and the office building B are showed in the following figures (see Fig. 16 and 17), respectively.

**Fig. 18:**
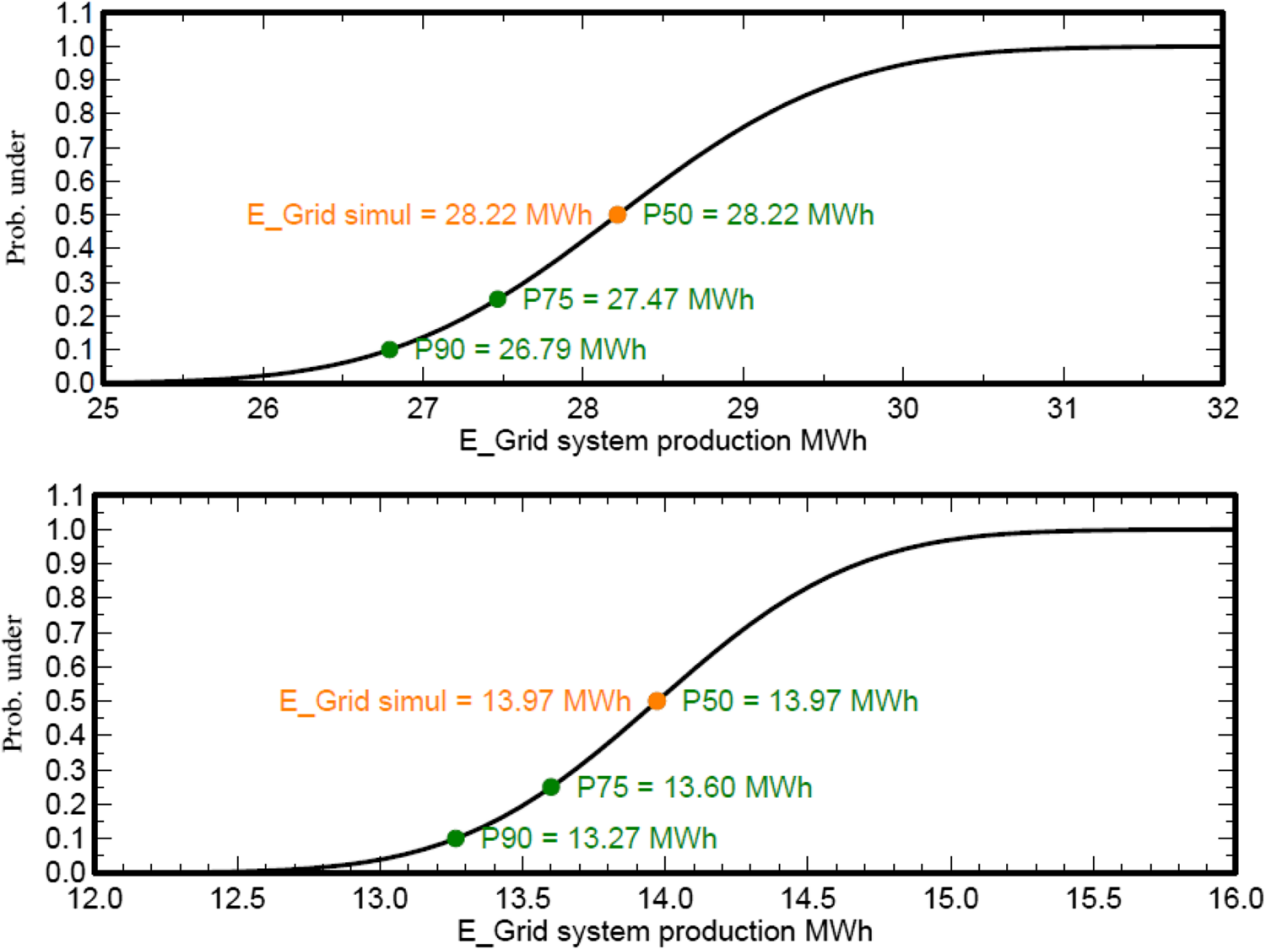
Repartition function of the control room building A and office building B on the top and bottom, respectively

**Fig. 19:**
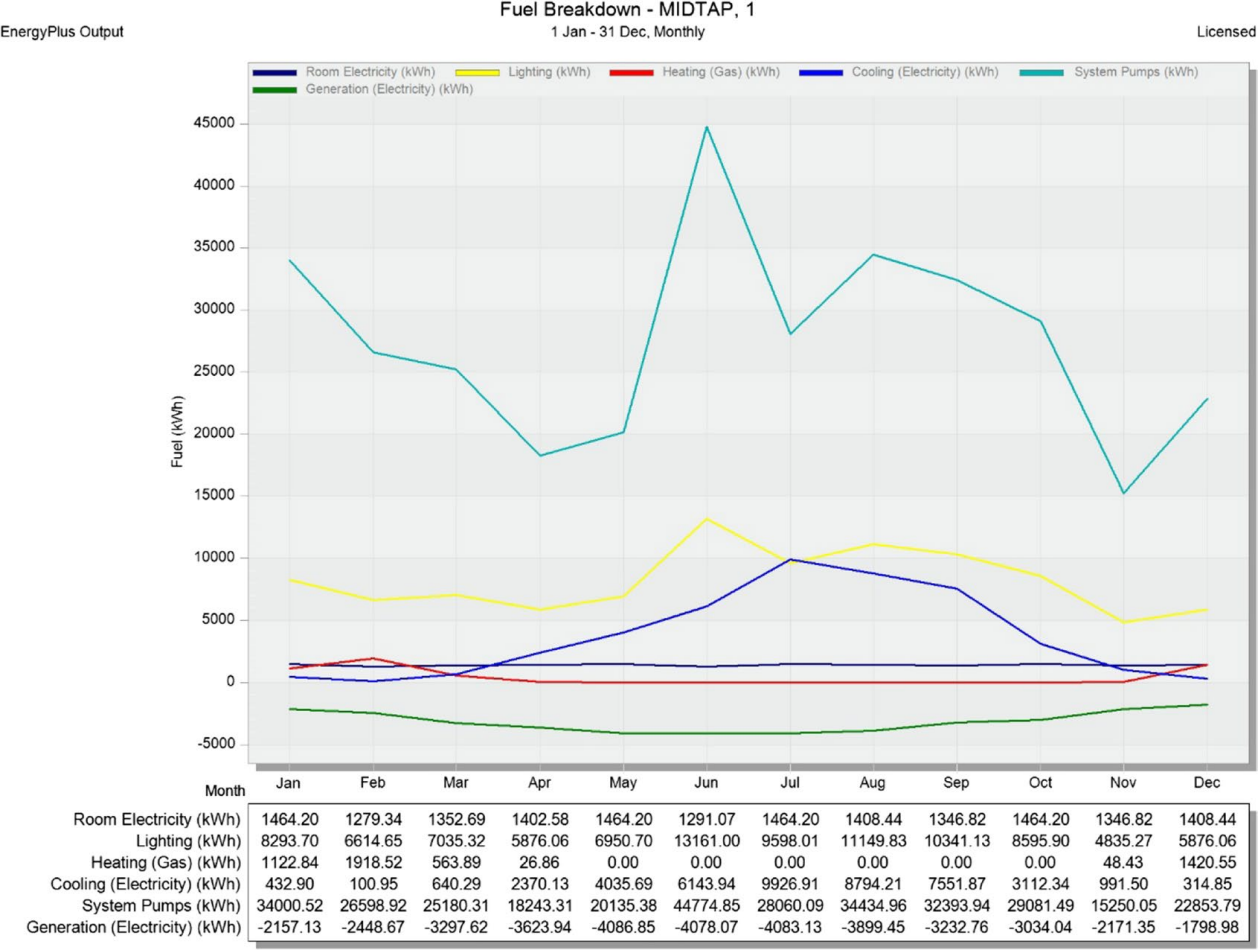
Fuel breakdown at the MIDTAP port through the whole year

Eventually, an evaluation of the production probability forecast is implemented for the grid-connected system. The probability distribution of the system production forecast for different years is mainly dependent on the input data used for the simulation. The annual production probability showed a variability of 1.11 MWh and 0.55 MWh of the control room building A and the office building B, respectively. As well, repartition function showed an energy of 28.22 MWh for the control room building A and 13.97 for the office building B (see Fig. 18). Fuel consumption in kWh describes the power required during the year for each month (see Fig. 19 and Table 5). Also, the fuel breakdown at the MIDTAP port is covered by the electricity generated by the solar system.

**Table 5:** Electricity generation and distribution

	Electricity	Percent Electricity
	[kWh]	[%]
Fuel-Fired Power Generation	0.000	0.00
High Temperature Geothermal*	0.000	0.00
Photovoltaic Power	28,165.513	24.49
Wind Power	11,154.765	9.70
Power Conversion	0.000	0.00
Net Decrease in On-Site Storage	0.000	0.00
Total On-Site Electric Sources	37,912.002	32.96
Electricity Coming From Utility	84,379.882	73.36
Surplus Electricity Going To Utility	7271.253	6.32
Net Electricity From Utility	77,108.629	67.04
Total On-Site and Utility Electric Sources	115,020.631	100.00
Total Electricity End Uses	115,020.631	100.00

### Wind energy

#### Numerical implications 

**Fig. 20:**
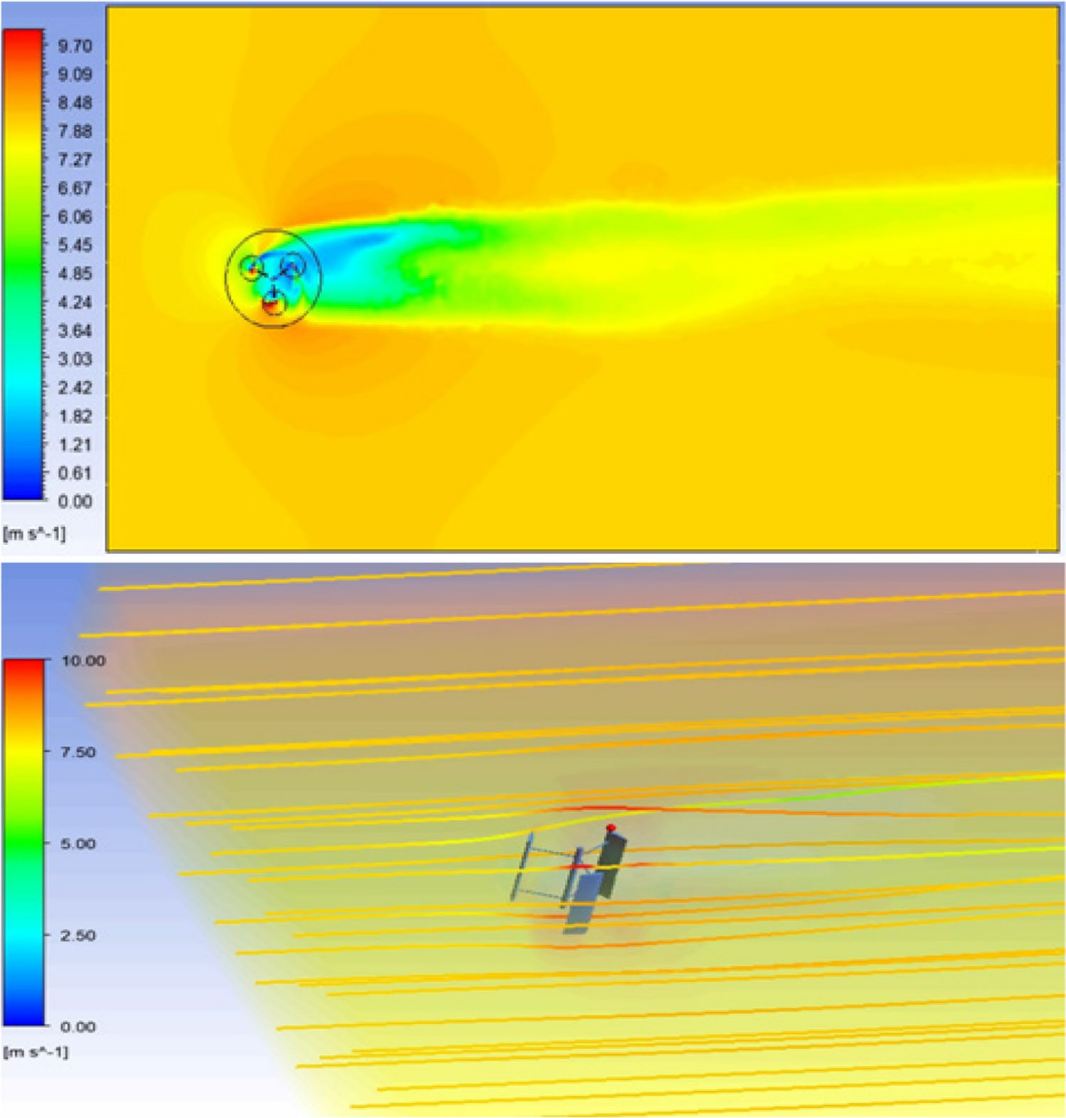
Velocity contours and streamlines for the VAWT on the building at the MIDTAP port on the top and bottom, respectively

The second part shows the results of the wind turbine simulations. The first simulation showed characteristics of the VAWT that indicated good aerodynamics coefficients. Velocity contour and streamlines of the air flow showed the rotating turbine interaction with air as illustrated in Fig. 20 on the top and bottom, respectively. The single wind turbine had a power coefficient of 0.123, which is a reasonable value regarding the same rotor boundary condition.

**Fig. 21:**
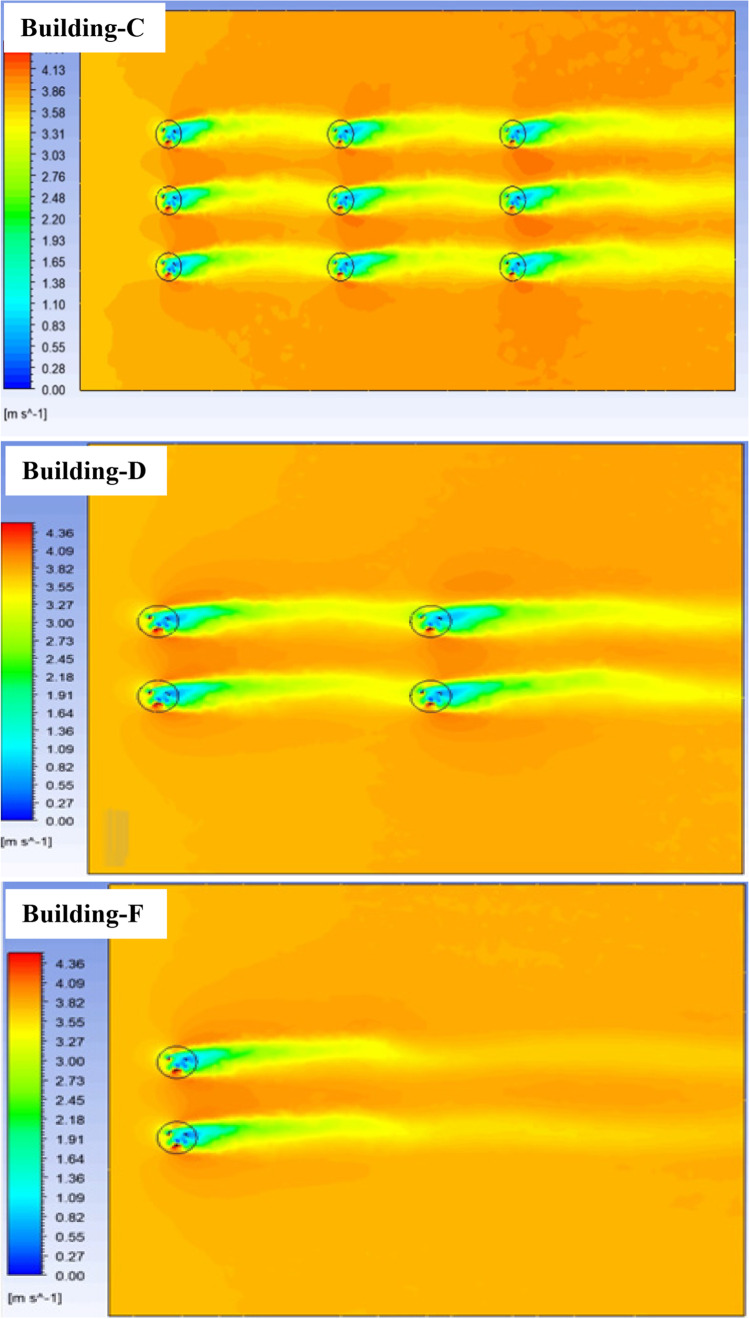
Velocity contours for arrays of VAWTs, arranged 3 × 3 in 1, 2 × 2 in 2, and 2 × 1 in 3 for building C, D, and F, respectively

Meanwhile, the arrays of VAWTs are simulated with the boundary condition of the MIDTAP port. Three cases showed good aerodynamics behaviors (see Fig. 21). The power coefficients of each rotated turbine are calculated and listed as shown in Table 6. However, these values increased for each turbine than the single turbine. These increases were noticed also in previous research due to the aerodynamics interactions (Xu et al. 2021). The increases in power coefficient indicate to enhance the overall array efficiency.

**Table 6:** Power coefficient of all VAWTs on buildings

Building	Power coefficient (Cp)			Increased percentage of Cp		
C	0.150	0.138	0.134	17.5%	7.9%	4.8%
	0.151	0.142	0.133	18.2%	11.1%	4.2%
	0.152	0.141	0.134	18.9%	9.7%	4.5%
D–E	0.149	0.137		16.7%	7.1%	
	0.151	0.144		18.2%	12.5%	
F	0.150			16.9%		
	0.151			17.6%		

### Energy generation and optimization

#### Policy recommendations 

**Fig. 22:**
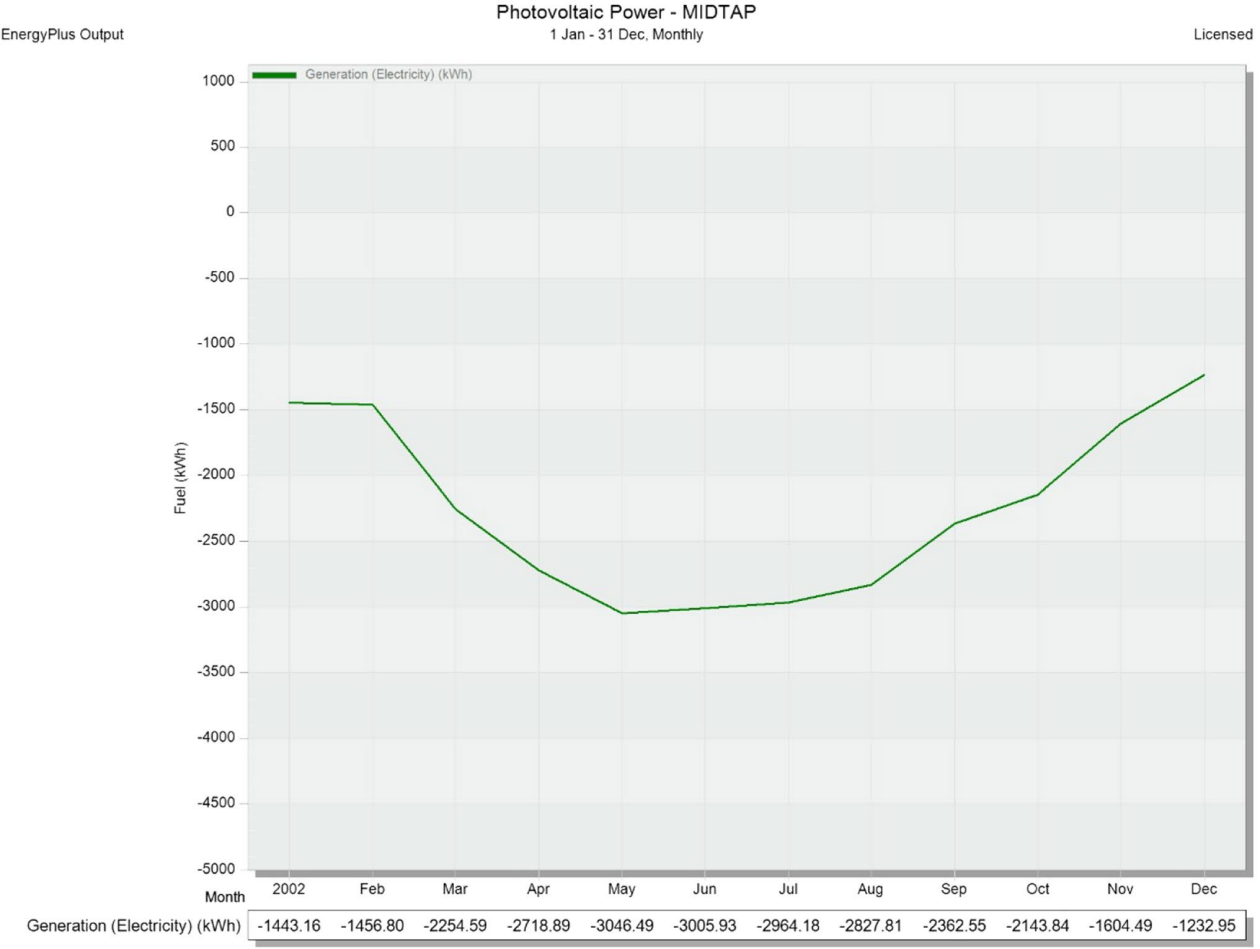
Monthly photovoltaic power generation (KWh) through a full year January 1–December 31 at the MIDTAP port

**Fig. 23:**
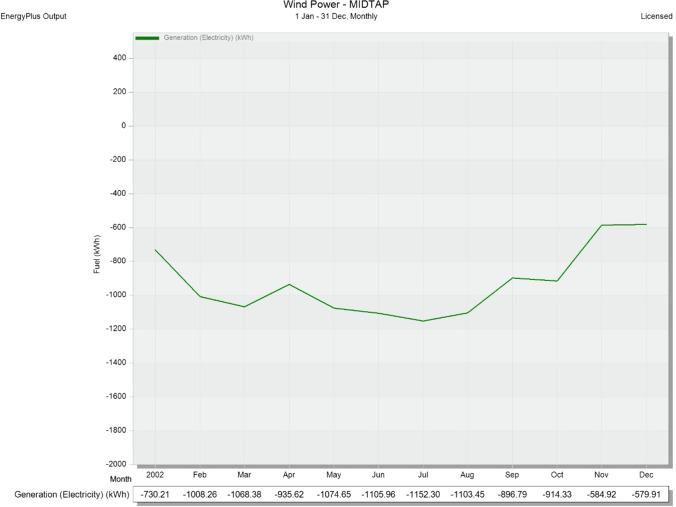
Monthly wind power generation (KWh) through a full year January 1–December 31 at the MIDTAP port

Optimization of the power generations’ usage is carried out based on the Pareto optimality analysis method for the port’s hybrid system. The analysis is used the given data by the power generations of photovoltaic systems and the wind turbines. Collected data from the installed weather station is fed to the DesignBuilder software. That data is used to simulate the solar energy generation with respect to the energy consumption by the port’s buildings and validated to the actual solar generated power. Therefore, the simulation resulted a monthly photovoltaic power generation (KWh) through a full year January 1–December 31 at the MIDTAP port (see Fig. 22). Meanwhile, the turbine power coefficients (see Table 6) are fed to the DesignBuilder software to simulate the wind energy generation this time with respect to the energy consumption by the buildings too. The results of the wind turbine power generation (KWh) are shown monthly through the same year of the solar generation (see Fig. 23).

Nevertheless, it is obvious that the power generation is increased during the summer months, starting from March, and then starts to decrease again by September and October (see Fig. 22). The maximum and minimum power generations through the whole year are 3046.5 KWh and 1232.9 KWh achieved on May and December, respectively. Meanwhile, the power generation of the wind turbines showed kind of different behavior than the solar generation. The generated power is maintained in a fixed range for the year, except for November and December (see Fig. 23). The maximum and minimum power generations are 1152.3 KWh and 579.9 KWh for months of July and December, respectively. All values are negative because it cut down the power consumption of the main grid.

Pareto optimality analysis between the power generations of photovoltaic systems and the wind turbines for the hybrid system is applied to evaluate the energy management plan. All port’s buildings consume power from the main national grid. However, solar energy and wind energy are providing a scheme to cover the used power, which would be a strategy of reducing these consumptions. This analysis consists of one objective function, which is selecting the best power generation method to cover up the power usage by the buildings on a large time scale. Pareto optimality solution investigated variations between the power generations of solar energy and wind energy over time.

**Fig. 24:**
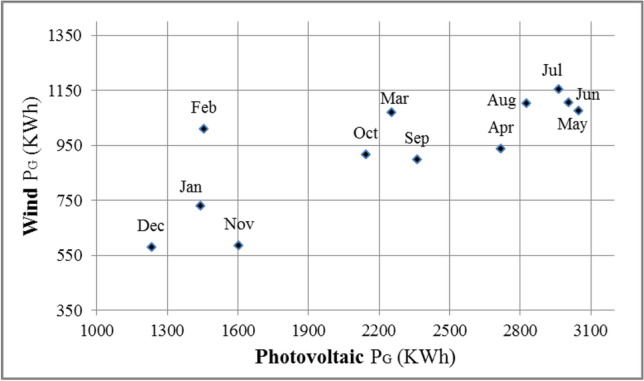
Pareto optimality analysis chart between the power generations of photovoltaic systems and the wind turbines for the port’s hybrid system

Figure 24 shows a chart of a simple Pareto solution revealing that the maximum power generation was on July, June, and May. December, November, and January had the lowest power generation, in which the rest of the required power from the main grid could be used. Therefore, the power consumed from the main grid could be reduced or even some of the generated renewable energy charges to the main grid during the summer months. However, during the winter months, the power generation sometimes may not be sufficient for all the port’s buildings, and the system will draw from the main grid unlike the summer months. Therefore, the power generations from the solar energy and wind energy will provide the required energy consumption for the port’s buildings for the rest of the year.

### Discussion of results

Due to the solar panels for both buildings have been installed, the total nominal output is 13.26 kWp at an operating condition of 11.82 kWp. The simulation showed that the buildings have saved energy consumption and reduced the emission of course by around 42.19 MW/year with an average of 72.25%. On the other hand, the system is providing around 42.191 MWh to be returned to the main national grid as an added value.

The wind system, on the other hand, has a great chance to be implemented in the future. The front area of the port has a good wind speed without any obstacles affecting the wind flow. With the good power coefficient of the wind rotor, the wind system will help the solar system to support all the port’s buildings, especially with the increased power coefficient as shown by the simulations. The wind system will be addition to the whole renewable energy system as the intuition of the port authority to go with the green seaport.

The DesignBuilder software showed the compatibility of both the wind and the solar systems together. Then, the Pareto optimality analysis method approved that by showing that the wind and solar systems can work simultaneously to provide green energy through the whole year. Power generation is increased during the summer months because the addition of the solar system and the increased efficiency of the PV panels.

To sum up, the uncertainty of the solar and wind energy is covered by using two renewable energy systems instead of one, plus an energy management plane, which manages the operation of both systems. However, the energy demand here is considered the energy required for the buildings. The DesignBuilder software is described and how the energy distribution through the whole year would be covered. That is the role of the energy management plan, which plays a critical role of providing the energy and ensure the reliability of the system. Mainly, based on the results of the DesignBuilder results, solar energy supports the system during the summer months, and wind energy supports the winter months. On the other hand, solar energy works only during the daylight; therefore, a storage system would help provide energy for the building during the nighttime, if needed. However, wind turbines provide energy during the whole day. Notice, the DesignBuilder software analyzed the energy input and output based on the given system, while the Pareto optimality method is used to evaluate the operation of the energy management plan.

Energy supply is the energy that could be provide by the national grid or could be provide by the solar and wind energy, in the case of green port. The seaport in that case uses the national grid for providing the energy needs for workshops, machineries, and buildings. However, the solar system is working properly to provide energy supply to the building and reduce the consumption of the national grid.

Based on the given case, the buildings demand energy daily, which is covered by the energy supply of the national grid. Adding renewable energy application to the port’s buildings made the port self-sufficient. However, the plan is to have a full coverage of the renewable energy to cover the energy demand. Thus, the energy supply of the port will have excess generated energy. That excess amount could be store into a battery system; however, the plan is to send it to the national grid, which will make profit and reduces the operational cost of the system.

## Conclusion and future works

Solar energy and wind energy have been applied at the port of Alexandria, practically and theoretically, respectively. At the meantime, no research tackles the problem in Egypt. Solar energy is investigated using onsite arrays, and then energy management is carried out to the installed system using the DesignBuilder software. The energy management also included wind energy, which is examined numerically, as an initial step. However, there is no study which investigated the optimum strategy for solar and wind energy in maritime ports. The solar panel experiment showed a sufficient generated power for all the port’s buildings, while the numerical model validated the real testing. Also, the wind arrays simulation had good behavior with power coefficient more than the single wind turbine, due to the array interaction. Finally, Pareto optimality analysis is carried out between the generated powers from solar and wind energies over the year to reveal an energy management plan for the power generation and the power usage of all buildings. Therefore, the solar energy and the wind energy will reduce the energy consumption of the port by reducing the energy consumption of the port’s buildings. Nevertheless, an onsite wind turbines array will be experimented as a future investigation to ensure the best design validation, while using machine learning code for an advanced optimization.

## Limitation of the study

The use of renewable energy application in a maritime port in Alexandria, Egypt, is a challenge. In this research, the use of an optimized management plane to operate the renewable energy application has been studied. Two numerical approaches are used to simulate the wind energy and the solar energy, while the solar energy has been applied onsite.

## Data Availability

The data that support the findings of this study are available from the corresponding author Ahmed S. Shehata, upon reasonable request. In addition, the experiment, devices, and software used are available in the renewable energy lab, in Marine Engineering Department, Arab Academy for Science Technology and Maritime Transport, P.O. 1029, Abu Qir, Alexandria, Egypt.

## Abbreviations

PM: Particle matter
GHG: Greenhouse gases
OPS: Offshore power supply
COE: Cost of energy
NPC: Net present cost
RF: Renewable fraction
PV: Photovoltaic
GSHP: Ground-source heat pumps
TSR: Tip speed ratio
SMM: Sliding mesh motion
CFD: Computational fluid dynamic
VAWT: Vertical axis wind turbine
LES: Large eddy simulation
